# The relative age effect is larger in Italian soccer top-level youth categories and smaller in *Serie A*

**DOI:** 10.1371/journal.pone.0196253

**Published:** 2018-04-19

**Authors:** Paolo Riccardo Brustio, Corrado Lupo, Alexandru Nicolae Ungureanu, Riccardo Frati, Alberto Rainoldi, Gennaro Boccia

**Affiliations:** 1 NeuroMuscularFunction | Research Group, School of Exercise and Sport Sciences, SUISM, Department of Medical Sciences, University of Torino, Turin, Italy; 2 School of Exercise & Sport Sciences, SUISM, University of Torino, Turin, Italy; Universita degli Studi di Verona, ITALY

## Abstract

The relative age effect (RAE; i.e., an asymmetry in the birth distribution) is a bias observed in sport competitions that may favour relatively older athletes in talent identification. Therefore, the aim of this study was to investigate the presence of RAE in elite soccer players competing in the Italian championships, even considering the discriminations of younger and older *Serie A* players (in relation to the median age of the sample), and different positional roles (i.e., goalkeeper, defender, midfielder, forward) for each observed category. A total of 2051 players competing into the 2017–2018 Italian under-15 (n = 265), under-16 (n = 362), under-17 (n = 403), *Primavera* (n = 421) and *Serie A* (n = 600) championships were analysed. The birth-date distributions, grouped in four quartiles (i.e., January-March, Q1; April-June, Q2; July-September, Q3; October-December, Q4), were compared to a uniform distribution using Chi-squared analysis. The week of birth was analysed using Poisson regression. The results showed a large over-representation of players born in Q1 in all soccer player categories. However, the effect size of this trend resulted smaller as age increased. Individuals born in Q1 have about two-folds more chances to become a *Serie A* player compared to those born in Q4. The Poisson regression analysis showed that RAE was greater for defenders than for forwards among all categories. Therefore, a strongly biased selection emerged among elite soccer players competing in Italian championships, highlighting how young individuals born in the first three months have many more chances to become elite players compared to the others.

## Introduction

In Italy, as in most other countries, adolescents and young athletes are grouped according to their year of birth. Such a categorization of athletes in age groups, based on their chronological age (e.g., considering as cut-off date January 1^st^), is an usual choice to arrange young athletes in sports competitions [1, 2]. Even though this process is necessary in terms of sport management [3], there is a difference of almost one year between athletes born close to (e.g. relatively older athletes) and far away (e.g. relatively younger athletes) the cut-off date and this could determine crucial consequences in sport career.

The age difference between athletes grouped in the same category (i.e. athletes born in the same calendar year) has an effect both in terms of physical and psychological maturation [1, 2]. Relatively older athletes are advantaged compared with relatively younger athletes in sport performance [4], and consequently in the process of sport talent identification [2, 3, 5]. In fact, the former subgroup of athletes is more likely to be selected by elite and national teams, and thus they are more likely to become a professional athlete [1, 2, 6]. Specifically, the athletes born close to the cut-off date are more likely to achieve success in sport activities [1]. Therefore, this asymmetry in the birth distribution is referred as Relative Age Effect (RAE) [7, 8], and it has been initially observed in Canadian ice hockey [7], and other individual [9–12] and team [2, 13–17] sports.

Similar to other sports, soccer is characterized by a significant over-representation of players born in the early part of the year among young athletes [2, 18, 19]. However, despite the pervasive presence of RAE in youth soccer teams, controversial results were found in senior categories. For example, González-Víllora and colleagues [13] found no evidence of RAE in the senior (professional) soccer teams, whereas other studies clearly showed the existence of this effect in the same category of athletes [20, 21].

For Canadian ice hockey [7] and Eighties’ elite soccer players [22], RAE incidence seemed to progressively decrease as the age category increased, highlighting stronger RAEs in youth players with respect to senior ones. In particular, having been classified as a strength-based sport, soccer teams benefit from optimal players’ physical characteristics such body size [23], aerobic power, muscular strength, endurance, and speed, which are considered as the most important factors to achieve success [1]. Therefore, in line with these soccer aspects, goalkeepers and defenders (more decisive in the defensive actions) could benefit from optimal physical characteristics, reporting higher RAEs with respect to midfielders and forwards. However, this hypothesis has been not fully confirmed by previous studies, where only elite Italian midfielders reported an over-representation of players born in the early part of the selection year [24], whereas no particular RAE divergence emerged between different tactical roles of the elite Spanish soccer [19].

In Italy, soccer is the most popular and played sport among young and adult males. Marketers, mass-media, and entrepreneurs invest a considerable amount of money in Italian soccer sponsorship and ownership. The topic of the soccer players’ RAE in Italian youth categories [2] and senior top professional league *(Serie A)* has been considered in previous studies [24, 25]. In particular, a study [2] showed an over-representation of players born in the first quartile of the selection year (from January to March) in Italian youth soccer categories (U-15, U-16, U-17, and U-18), whereas other two works [24, 25] reported the same effect for *Serie A* players, even highlighting effects in terms of gross wages [24] and tactical roles [25].

However, to the best of our knowledge, no study provided evidence of the RAE for Italian soccer players of each youth and senior age category of *Serie A* clubs and among tactical roles. Thus, this study aimed to investigate: i) the RAE in soccer players of each age category (i.e., Under 15, U15; Under 16, U16; Under 17, U17; Under 20, *Primavera*; and senior top professional league, *Serie A*) of clubs competing in *Serie A* (i.e., the most prestigious senior Italian soccer championship); ii) even considering the comparison between younger and older *Serie A* players’ subgroups established in relation to the median age of the sample; and iii) analyzing if RAE differences can emerge in relation to different positional roles (i.e., goalkeeper, defender, midfielder, forward) in each age category. In particular, according to literature on RAE, it has been hypothesized that: i) elite soccer players competing in Italy and born in the early part of the selection year would be over-represented; ii) RAE would be present in both youth and senior categories, confirming that this trend is not attenuated in the older athletes; and iii) that the positional role would not influence the size of RAE.

## Materials and methods

### Participants

To assess the prevalence of RAEs in players competing in the Italian soccer championships, a substantial data set has been collected from the web sites of *Serie A* clubs. The sample consists of 2064 soccer players competing in the Italian 2017–2018 championships. In particular, according to the official web site of the Italian Football Federation (http://www.campionatigiovanili.it/), U15 (n = 265), U16 (n = 362), and U17 (n = 416) were considered as youth categories, whereas players related to *Primavera* (n = 421) and *Serie A* (n = 600) were included in the study as senior categories.

The study was conducted in accordance with the declaration of Helsinki. Because these data are based on anonymous resources and publicly available information, no informed consent was requested. However, this study was approved by the local ethics committee of the University of Torino (Turin, Italy).

### Procedure

Young and professional soccer players selected for this study were classified in relation to their birth-date and positional roles. Successively, all athletes were categorized in four groups based on their month of birth. Specifically, players born between January and March, April and June, July and September, and October and December were classified into the quartile 1 (Q1), quartile 2 (Q2), quartile 3 (Q3), and quartile 4 (Q4), respectively.

Even though the Italian nationality was the most represented in the sample (e.g., 45.4% in *Serie A*; www.transfermarkt.it/serie-a), a substantial portion of players selected for the present study were foreign. Thus, according with Delorme and Champely [26] we decide to compare the distribution of players' birthdates with a uniform distribution (i.e., Q1 = 25%; Q2 = 25%; Q3 = 25%; Q4 = 25%). This approach is frequently adopted in the literature for the RAE assessment of international samples.

Furthermore, it has been explored the possibility that the RAE might be more evident in younger than older *Serie A* players. For this purpose, the birth-date distributions of two sub-groups of *Serie A* players were compared, discriminating participants in relation to the median age of the sample.

### Statistical analysis

Chi-square (χ^2^) tests for U15, U16, U17, *Primavera*, and *Serie A* categories were carried out to investigate the difference in birth-date distribution in the Q1, Q2, Q3, and Q4 quartiles compared to a uniform distribution. In addition, the same statistical approach has been provided in relation to each positional role (i.e., goalkeeper, defender, midfielder, forward), and for the younger and older *Series A* players’ subgroups (after calculating their median age).

For all analyses, the effect size ω of the Chi-square tests was calculated according to Cobley and colleagues [1] and Brazo-Sayavera and colleagues [9]. In addition, odds ratios (ORs) and 95% confidence intervals (95% CI) were calculated for the first and the last quartile (i.e., Q1 and Q4), as well as for the first and the second semester of the year (i.e., Q1 + Q2 and Q3 + Q4).

Despite the use of Chi-square tests to assess the RAE has been widely adopted [9, 11, 13, 15], such method has been criticized because it is considered to have a low statistical power and to be vulnerable to non-RAE signature [27], making as necessary the application of the Poisson regression for analyzing low count data [28]. The formula of the Poisson Regression y = e^(b0 + b1x)^ allows to explain the frequency count of an event (y) by an explanatory variable x. As consequence, the week of born (W_B_) of each player was assessed as follows: players born between 1^st^ and 7^th^ January were categorized in W_B_ 1, players born between 8^th^ and 14^th^ January were categorized in W_B_ 2 and so on.

The time of birth (t_B_) was computed according to the formula t_B_ = (W_B_ − 0.5) / 52. In particular, t_B_ measures how far from the beginning of the year a player was born, and it is ranged between 0 and 1. Successively, using a Poisson regression to count data, it has been calculated how the frequency of birth in a given week (y) was explained by the t_B_ (x). Additionally, according to Doyle and Bottomley [28], the Index of Discrimination (ID) was calculated as e^-b1^. The ID provides the relative odds of being selected for a player born on day 1 versus day 365 of the competition year [28]. Likelihood ratio R^2^ was computed according to Cohen and colleagues [29].

Statistical analyses were conducted using Statistical Package for Social Sciences, version 24.0 (SPSS Inc, Chicago, IL, USA) and GraphPad Prism (San Diego, California, USA). For all tests, the level of significance was fixed at α ≤ 0.05.

## Results

**Fig 1:**
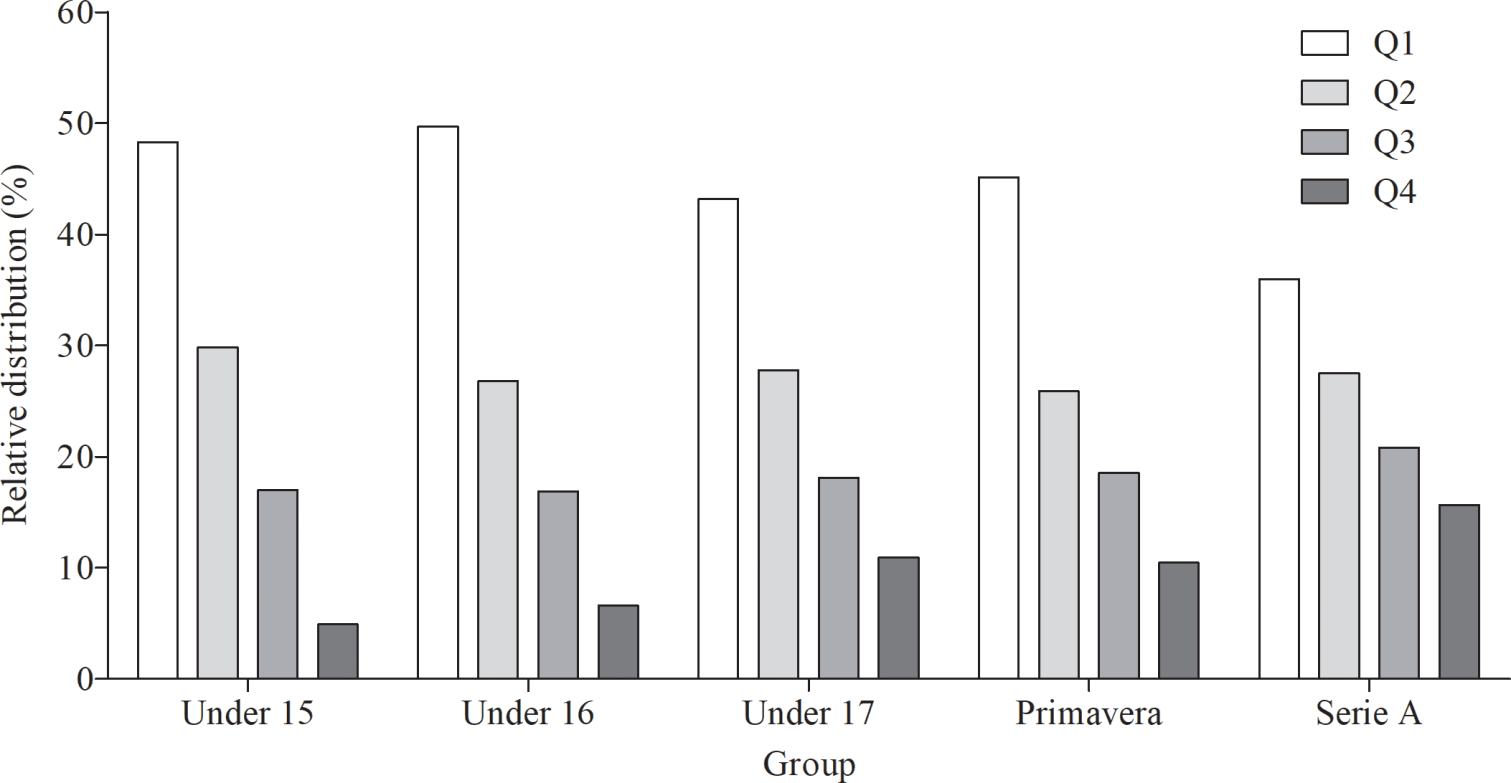
Birth date distribution of soccer players competing in the Italian championship. Quarterly birth date distribution of soccer players competing in the Italian U15, U16, U17, *Primavera*, and *Serie A*. The uniform distribution was of 25% for each quartile. Quartile 1 (Q1), 2 (Q2), 3 (Q3), 4 (Q4).

The observed quarterly birth rate distribution of the participants for U15, U16, U17, *Primavera*, and *Serie A* is separately displayed in Fig 1.

Table 1 shows the pertinent statistical analysis in relation to the overall sample and different positional roles (i.e., goalkeeper, defender, midfielder, forward).

**Table 1:** Unadjusted odds ratios (ORs, 95% CI) for soccer players examining relative age effect.

		Under 15	Under 16	Under 17	*Primavera*	*Serie A*
**Overall**	χ^2^	60.5	74.4	43.9	53.5	27.7
	*P* value	<0.0001	<0.0001	<0.0001	<0.0001	<0.0001
	**ω**	**0.47**	**0.45**	**0.33**	**0.36**	**0.22**
	OR Q1/Q4	9.8 (5.1–19.1)	7.5 (4.5–12.6)	4.0(2.6–6.1)	4.3 (2.8–6.6)	2.3 (1.6–3.2)
	OR (Q1+Q2)/(Q3+Q4)	3.6 (2.4–5.2)	3.3 (2.4–4.5)	2.4 (1.8–3.3)	2.5 (1.8–3.3)	1.7 (1.4–2.1)
**Goalkeeper**	χ^2^	10.64	8.7	11.6	7.42	8.4
	*P* value	0.0138	0.034	0.0091	0.0596	0.0392
	**ω**	**0.68**	**0.50**	**0.51**	**0.31**	**0.35**
	OR Q1/Q4	31 (1.5–634.9)	9.5 (1.7–53.4)	21 (2.4–18436)	4.4 (1.2–15.6)	4.0 (1.5–11.0)
	OR (Q1+Q2)/(Q3+Q4)	4.8 (1.2–18.2)	3.4 (1.2–9.4)	2.4 (1–5.7)	1.6 (0.7–3.8)	2.2 (1.1–4.3)
**Defender**	χ^2^	24.0	23.2	18.6	17.3	7.3
	*P* value	<0.0001	<0.0001	0.0003	0.0006	0.006
	**ω**	**0.59**	**0.47**	**0.38**	**0.37**	**0.19**
	OR Q1/Q4	19.5 (4.0–94)	10.2 (3.5–29.7)	5.1 (2.3–11.5)	4.5 (2.0–10)	2.0 (1.2–3.7)
	OR (Q1+Q2)/(Q3+Q4)	5.9 (2.6–13.4)	3.2 (1.7–5.7)	2.8 (1.6–4.7)	2.8 (1.6–4.7)	1.7 (1.2–2.5)
**Midfielder**	χ^2^	17.2	25.7	13.0	18.7	8.7
	*P* value	0.0006	<0.0001	0.0047	0.0003	0.0332
	**ω**	**0.49**	**0.49**	**0.31**	**0.35**	**0.21**
	OR Q1/Q4	12 (3.1–46.2)	7.7 (3.0–20)	2.4 (1.2–4.9)	4.5 (2.2–9.2)	2.2 (1.3–3.9)
	OR (Q1+Q2)/(Q3+Q4)	3.2 (1.6–6.6)	4.2 (2.3–7.7)	2.5 (1.5–4.1)	2.3 (1.4–3.6)	1.7 (1.2–2.6)
**Forward**	χ^2^	6.6	13.9	12.1	14.6	5.5
	*P* value	0.0868	0.0031	0.0071	0.022	0.1404
	**ω**	**0.36**	**0.39**	**0.35**	**0.38**	**0.20**
	OR Q1/Q4	3.6 (1.0–12.6)	5.4 (2.1–13.9)	4.2 (1.8–9.9)	3.9 (1.7–9.1)	2.1 (1.1–4.2)
	OR (Q1+Q2)/(Q3+Q4)	2.7 (1.2–6.2)	2.2 (1.2–4.1)	2.2 (1.2–3.8)	2.9 (1.6–5.5)	1.7 (1.0–2.7)

Quartile 1 (Q1), 2 (Q2), 3 (Q3), 4 (Q4).

The observed distributions for U15, U16, U17, *Primavera*, and *Serie A* were significantly different from the uniform distribution (all *P* values < 0.0001). Specifically, an over-representation of soccer players born in Q1 (overall mean 43.3%) and Q2 (overall mean 27.4%) was observed. Differently, an under-representation of players born in Q3 (overall mean 18.6%) and Q4 (overall mean 10.7%) was reported. Interestingly, the effect size of the RAE (as well as the ORs) resulted lower for higher progressive age categories, with the exception of *Primavera*, which reported a little higher value with respect to the U17 one.

Overall, the positional role did not influence the effect size of the RAE, with the exception of U15. Indeed, when considering the ω and ORs, the U15 goalkeepers and defenders showed larger RAE than midifielders and forwards (Table 1). All other age categories did not show marked difference among tactical roles (Table 1).

The calculation of the median age of the Italian *Serie A* soccer players defined < and ≥ 25 years old players as younger and older, respectively. For the RAE distribution of younger and older *Serie A* players, differences emerged with respect to the uniform distribution for both subgroups (Table 2). Nevertheless, no difference was reported in comparing the RAE of younger and older subgrougs (χ^2^ = 2.1, *P* = 0.532, Q1/Q4 OR 1.2, CI0.7–2.0, OR (Q1+Q2)/(Q3+Q4) 1.0, CI 0.7–1.4.

**Table 2:** Relative age effect (RAE) differences between younger and older soccer players playing in *Serie A*.

*Serie A*		Younger (<25 yrs)	Older (≥25 yrs)
	χ^2^	16.2	12.7
	*P* value	0.0011	0.0054
	**ω**	**0.23**	**0.20**
	OR Q1/Q4	2.6 (1.6–4.2)	2.1 (1.3–3.3)
	OR (Q1+Q2)/(Q3+Q4)	1.8 (1.3–2.5)	1.8 (1.3–2.5)

**Fig 2:**
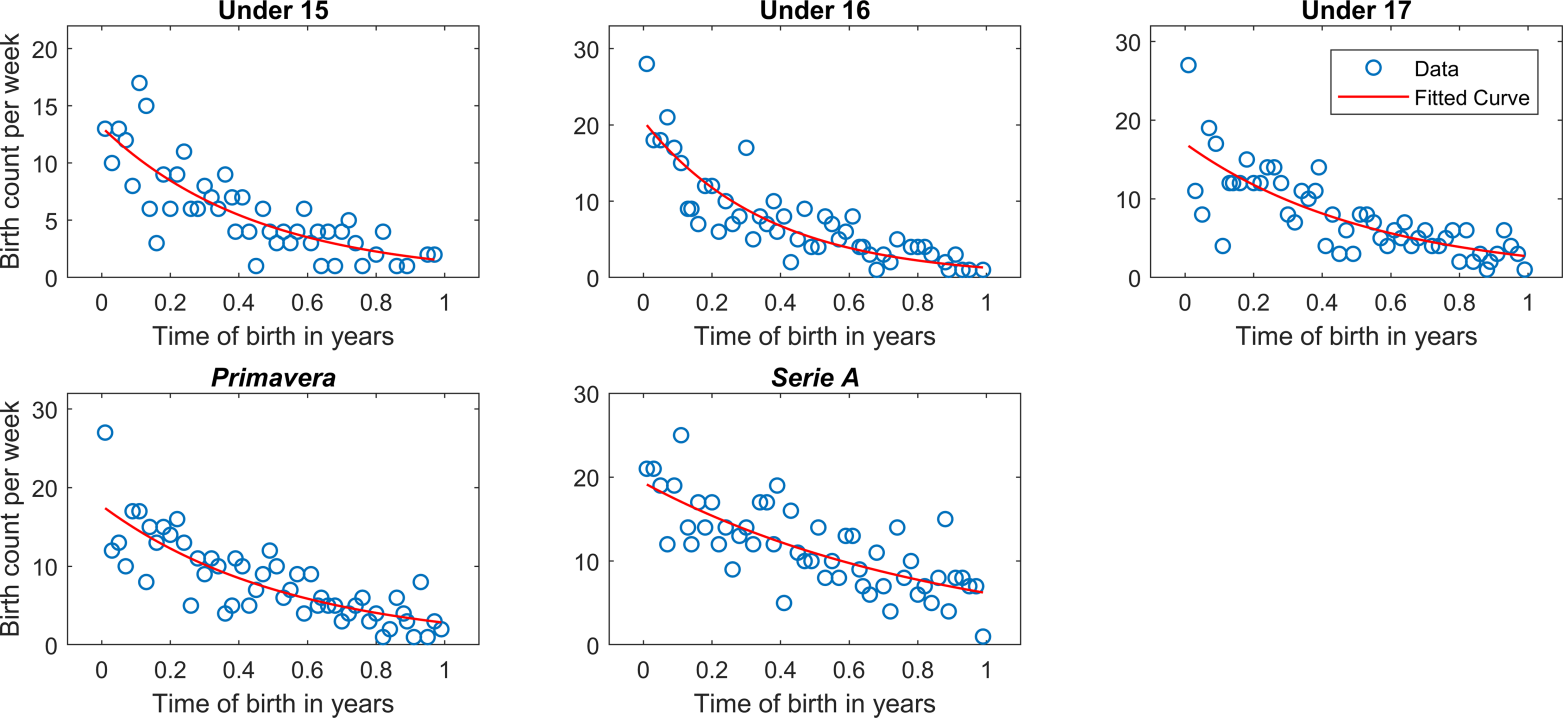
Scatterplot of RAE frequency by week. Scatterplot of RAE frequency by week of year for U15, U16, U17, *Primavera*, and *Serie A*. The red line represents the best fit of the Poisson regression.

Table 3 shows the mean and standard deviation of W_B_ and t_B_, as well as the results of Poisson regression for each category. The scatterplot of relative birth frequency by week is reported in Fig 2. The findings from this analysis were in general similar to those coming from the Chi-squared analysis. Indeed, in general, the frequency of t_B_ was higher at the beginning of the year compared to the end of the year. Furthermore, ID showed that the players born right at the start of the year were 8.93 times more likely to be included in Under 15 than those born at the end of the years, 11.88 times more likely to be included in Under 16, 9.47 times more likely to be included in Under 17, 9.47 times more likely to be included in *Primavera*, and only 3.12 times more likely to be included in *Serie A*.

**Table 3:** Relative age effect (RAE) according to the Poisson regression in each category and positional role.

		Under 15	Under 16	Under 17	*Primavera*	*Serie A*
**Overall**	W_B_	16±14	16±13	19 ± 14	19 ± 14	22 ± 15
	t_B_	0.30 ± 0.27	0.30 ± 0.25	0.35 ± 0.26	0.36 ± 0.27	0.41 ± 0.28
	b_0_	2.573	2.954	2.989	2.87	2.961
	b_1_	-2.189	-2.475	-2.248	-1.840	-1.137
	**ID**	**8.93**	**11.88**	**9.47**	**6.30**	**3.12**
	R^2^	0.67	0.76	0.66	0.64	0.53
	*P* value	< 0.0001	< 0.0001	< 0.0001	< 0.0001	< 0.0001
**Goalkeeper**	W_B_	13 ± 10	16 ± 13	17 ± 12	19 ± 14	19 ± 3
	t_B_	0.23 ± 0.20	0.29 ± 0.24	0.32 ± 0.24	0.35 ± 0.26	0.35 ± 0.27
	b_0_	0.548	0.676	0.788	0.987	1.092
	b_1_	-0.969	-0.679	-0.867	-0.912	-1.086
	**ID**	**2.64**	**1.97**	**2.38**	**2.49**	**2.96**
	R^2^	0.30	0.11	0.12	0.28	0.22
	*P* value	0.366	0.327	0.085	0.090	0.023
**Defender**	W_B_	14 ± 11	16 ± 13	18 ± 13	19 ± 14	22 ± 15
	t_B_	0.26 ± 0.22	0.30 ± 0.24	0.34 ± 0.26	0.36 ± 0.26	0.42 ± 0.28
	b_0_	1.431	1.617	1.621	1.588	1.771
	b_1_	-1.75	-1.837	-1.476	-1.481	-0.948
	**ID**	**5.76**	**6.28**	**4.37**	**4.40**	**2.58**
	R^2^	0.54	0.43	0.38	0.43	0.22
	*P* value	0.002	< 0.0001	< 0.0001	< 0.0001	< 0.0001
**Midfielder**	W_B_	17 ± 13	16 ± 12	20 ± 15	19 ± 14	22 ± 15
	t_B_	0.32 ± 0.25	0.29 ± 0.24	0.38 ± 0.28	0.36 ± 0.27	0.41 ± 0.28
	b_0_	1.247	1.70	1.620	1.757	1.780
	b_1_	-1.378	-2.026	-1.231	-1.405	-0.886
	**ID**	**3.97**	**7.58**	**3.43**	**4.07**	**2.43**
	R^2^	0.40	0.52	0.37	0.42	0.23
	*P* value	0.004	< 0.0001	< 0.0001	< 0.0001	0.001
**Forward**	W_B_	20 ± 13	18 ± 14	19 ± 14	19 ± 14	22 ± 15
	t_B_	0.37 ± 0.24	0.33 ± 0.27	0.35 ± 0.26	0.35 ± 0.27	0.41 ± 0.28
	b_0_	0.754	1.271	1.445	1.374	1.419
	b_1_	-0.681	-1.188	-1.363	-1.169	-0.892
	**ID**	**1.98**	**3.28**	**3.91**	**3.22**	**2.44**
	R^2^	0.11	0.17	0.30	0.21	0.20
	*P* value	0.218	0.008	< 0.0001	0.004	0.004

Notes: W_B_, week in which players born; t_B_, time of birth; ID, Index of Discrimination.

The position role had affected the amount of RAE in all categories. In fact, the Poisson regressions were not significant (P ≥ 0.09, R^2^ ≤ 0.30) in goalkeepers from under 15 to *Primavera*. However, in goalkeepers the results were likely affected by the small sample sizes. Indeed, t_B_ indexes were low (≤ 0.30) for all categories, suggesting the times of birth were markedly skewed towards the first part of the year. Beyond this, IDs were overall higher in defenders (ranged 4.37–6.28) than in forwards (ranged 1.98–3.91). The IDs of midfielders showed more scattered results (ranged 2.43–7.58).

## Discussion

The aim of this study was to examine the incidence of RAE in elite Italian soccer clubs, both for young and senior soccer players. For this purpose, the birth-date distribution of players competing in *Serie A* clubs has been investigated considering both senior and youth categories. A large over-representation of athletes born close to the cut-off date (i.e., the beginning of the calendar year) strongly emerged in youth teams. Coherently, this trend is maintained in senior elite teams of *Serie A*, though to a less extent, accepting the first hypothesis.

A very large RAE was found in all considered youth categories (i.e., U15, U16, U17) and *Primavera* (Figs 1 and 2). In fact, these results revealed a biased distribution [19] in Italian soccer leagues with an over-representation of athletes born in the first part of the calendar year. Similar results were previously found in youth soccer categories in European context [2, 13, 15, 18–21]. RAE in team sports is influenced by different factors, including physical, psychological, and environmental aspects, or by their combination [1, 6, 8, 30]. In particular, in invasion sports, such as soccer, the physical maturation of individuals influences the muscular strength, endurance, and speed, which are important factors for a successful performance [1, 8]. Thus, relatively older young soccer players are favoured due to their advanced anthropometric development (e.g., body mass, stature) and a superior soccer-specific strength and endurance compared with their younger counterparts [31, 32]. These findings underline that the differences in maturity status within age groups negatively affects talent identification due to the greater chance for relative older athletes to be selected. In this sense, more opportunities to play, practise, and train are provided to young athletes with a more advanced physical maturity. Consequently, they might have more chances to become elite senior athletes.

A marked RAE is still present in elite senior players of *Serie A* teams (Figs 1 and 2). This is evidenced by both the Chi-squared (Table 1) and Poisson regression analysis (Table 2). The Poisson regression has been recently proposed as a powerful statistical mean to assess the RAE [28]. Thanks to this analysis it has been possible to calculate that the players born close to the beginning of the year are 3.1 times more likely to play in *Serie A* than the players born close to the end of the year, as highlighted by the corresponding ID value in Table 2. This means that the biased selection of talented players in youth categories has an effect even in senior teams, thus highlighting that athletes born close to the beginning of the calendar year are more likely to achieve success in adult sport compared to the relatively younger ones [1]. Therefore, this trend appears as controversial with respect to what has been described in a recent review on RAE in youth and elite soccer [33], which highlighted that no significant RAE was found in elite soccer teams due to the fact that physical advantages do not exist in older players.

In addition, the absence of differences for the prevalence of RAE of younger (<25 years) and older (≥25 years) *Serie A* players strengthened how such trend is not attenuated in older athletes, confirming what has been reported in the second hypothesis. In other words, the initial selection of *Serie A* players, which is biased by the RAE, results in a long-time effect, and the individuals born in the second part of the calendar year show fewer chances to become *Serie A* players even in later part of their career. Moreover, the extent of RAE in senior teams was much smaller than in youth categories. It is likely that for the athletes born late in the year, the chance to be identified and selected as elite increases as the age increases. Indeed, the small difference in age and, as a consequence, in maturation status, has a smaller effect in physical performance of adult players. Finally, it must be considered that the massive presence of foreign players in senior teams could potentially determine a relevant influence on the RAE of the *Serie A* soccer players. In fact, the extent of RAE differs across countries because of physical and socio-cultural factors [6, 34].

The players born in Q1 are 10-folds more represented than those born in Q4 in U15 category (as reported by the Q1/Q4 ORs in Table 1). This ratio progressively decreases as age increases, but remains very large across all youth categories, confirming the same trends already reported for Canadian ice hockey [7] and Eighties’ elite soccer players [22]. Indeed, in *Primavera* the players born in Q1 are 4-folds more represented than those born in the Q4. The same trend, i.e. RAE decreases as age increases, was evidenced by the Poisson regression analysis (as reported by the IDs in Table 2). The larger RAE, observed in the pubertal phase, supports the maturation-selection hypothesis [1, 9]. This means that the biased selection of young talented players is much more evident in the early adolescence where any little inter-individual age difference can determine a substantial difference in terms of body maturation.

The findings regarding the positional role are more difficult to interpret, mostly because the small sample sizes available for some roles (e.g. goalkeepers) made the analysis less statistically powerful. Nevertheless, for the U15 league, the RAE in goalkeepers and defenders was larger than that in midfielders and forwards (Tables 1 and 2), thus suggesting that the more pronounced body maturation of early-born players has more positive effect for athletes that play closer to the opponents’ soccer goal. Regarding the other categories (i.e. U16, U17, *Primavera*, and *Serie A*), the results would be slightly different if considering Poisson regression or Chi-squared analysis. Indeed, IDs coming from the Poisson regression analysis are consistently higher for defenders than for forwards (Table 2). This means that, in line with a previous study on elite Italian soccer players [25], the soccer players’ body size could be associated to RAE and coaches’ selection, regardless of references to real performance [23]. Conversely, the Chi-squared analysis did not show a clear trend in differentiating the position roles among age categories (Table 1). A systematic comparison between the two statistical approaches is beyond the scope of this study though. However, since the Poisson regression has been suggested to have more statistical power than Chi-squared analysis, we can conclude that the size of RAE was greater in defenders than in forwards in all categories.

The results of this study showed how RAE is a relevant factor in reaching success in Italian soccer competitions. This process leads to a vicious circle where early-born children have an increased and persistent advantage over late-born children in sports [2]. Consequently, the selection bias associated with the RAE should be taken into account for talent identification (e.g., knowledge of birthdates of the players or ordered of shirt numbers according to player’s age) [35].

Some limitations of this study should be underlined. First, we collected players’ information at the beginning of the season (i.e., at the end of August 2017), which may be not fully representative of the entire competitive period (i.e. August 20^th^, 2017 –May 20^th^, 2018). In fact, several players in *Serie A*, moved to different clubs during a specific winter period dedicated to these exchanges, determining heterogeneous team rosters along the same season. Second, the present data refer to only one season, making difficult to understand if the RAE is getting worse over time. Therefore, further studies should provide wider scenarios able to show data from different Italian soccer categories along several seasons. Moreover, the present study focused only on male athletes, highlighting the need to investigate the possible gender difference in the Italian context, in line with previous studies [9, 36]. Then, to assess the players’ RAE, it has been only considered the birthdate, avoiding any reference to the individual performance (e.g., number of the match involvement or total minutes played during a season) [17] or salary engagements [24], highlighting how this additional information might be useful to better describe RAE.

For all these reasons, similar analyses in different countries should be promoted. In fact, controversial effects could be unexpectedly observed in relation to different factors such as the players’ earnings [18], and salaries [37]. As consequence, any discordance of results could encourage future investigations on potential training and selection aspects that affect RAE mechanisms.

In terms of methodology, youth players who participated in two championships were considered twice (as different individuals), despite they represented the 4.3% of all selected youth soccer players. Moreover, the data analyses were applied considering the reference to the Italian population, despite the relevant presence of non-Italian players, who probably experienced different age-category cut-offs along a different talent process. Finally, restricted U15 (216 over 265 players; 81.5% of all players selected for this subcategory), U16 (241 over 265 players; 93.4%), and *Primavera* (599 over 600 players; 99.9%) samples were considered for the analysis on RAE in relation to tactical roles, due to lack of information.

In conclusion, it has to highlight that this is only the second paper which applied the Poisson regression [28], and how future studies on this topic will be able to consolidate this statistical procedure as more statistically power than Chi-squared analysis to elaborate RAE effects on the concept of Index of Discrimination.

## Conclusions

The birth-date distribution of young soccer players clearly showed a large over-representation of athletes born in the first semester of the year (i.e., Q1 and Q2). This trend is maintained, to a less extent, in senior elite teams of *Serie A*. Therefore, in Italian soccer context, the relatively older individuals have more chances to be selected by elite teams, both in young and senior categories. In fact, this selection bias, known as RAE, limits the possibility to potentially select talented athletes born late in the year of consideration. In addition, this selection bias could be influenced by the positional role, being greater in defenders than in forwards.

In conclusion, especially during the first years of soccer practice, coaches and physical trainers of soccer clubs should be aware that the players, born at the ending months of a year, even though are characterized by a not so successful performance (as consequence of lower values in body parameters, such as stature and body mass, as well as reduced physical and technical capabilities), could demonstrate high improvements for these aspects even in a close future. Therefore, the avoiding of this bias selection could effectively promote the grown of an entire national soccer system.

## Supporting information

S1 Table Data from participants in this study. (PDF)
