# Supplementary material for: The relative age effect is larger in Italian soccer top-level youth categories and smaller in Serie A
Source: PLoS One. 2018 Apr 19;13(4):e0196253. doi: 10.1371/journal.pone.0196253 (PMC5909613; doi:10.1371/journal.pone.0196253)
Supplement: S1 Table — (PDF) [file pone.0196253.s001.pdf]

DAY	MONTH	YEAR	ROLE	CATEGORY
2	1	2002	Defender	U15
2	1	2002	Defender	U15
9	1	2003	Defender	U15
13	1	2003	Defender	U15
13	1	2002	Defender	U15
18	1	2003	Defender	U15
24	1	2001	Defender	U15
27	1	2001	Defender	U15
30	1	2003	Defender	U15
2	2	2001	Defender	U15
7	2	2003	Defender	U15
7	2	2001	Defender	U15
8	2	2003	Defender	U15
10	2	2002	Defender	U15
25	2	2001	Defender	U15
5	3	2003	Defender	U15
17	3	2003	Defender	U15
19	3	2003	Defender	U15
25	3	2001	Defender	U15
13	4	2003	Defender	U15
14	4	2001	Defender	U15
22	4	2002	Defender	U15
3	5	2003	Defender	U15
6	5	2003	Defender	U15
24	5	2001	Defender	U15
23	6	2002	Defender	U15
25	6	2003	Defender	U15
13	7	2003	Defender	U15
28	8	2002	Defender	U15
29	9	2001	Defender	U15
27	10	2003	Defender	U15
6	1	2003	Defender	U15
11	1	2002	Defender	U15
11	1	2003	Defender	U15
17	1	2003	Defender	U15
18	1	2002	Defender	U15
23	1	2002	Defender	U15
26	1	2002	Defender	U15
26	1	2003	Defender	U15
6	2	2002	Defender	U15
10	2	2003	Defender	U15
14	2	2002	Defender	U15
14	2	2003	Defender	U15
14	2	2003	Defender	U15
16	2	2002	Defender	U15
18	2	2003	Defender	U15
20	2	2002	Defender	U15
8	3	2003	Defender	U15
11	3	2002	Defender	U15
18	3	2003	Defender	U15
25	3	2003	Defender	U15
11	4	2003	Defender	U15
18	4	2002	Defender	U15
20	4	2003	Defender	U15
25	4	2002	Defender	U15
25	4	2002	Defender	U15
29	4	2003	Defender	U15
9	5	2003	Defender	U15
14	5	2003	Defender	U15
19	5	2002	Defender	U15
27	5	2002	Defender	U15
30	5	2003	Defender	U15
23	6	2003	Defender	U15
6	7	2002	Defender	U15
15	7	2003	Defender	U15
14	8	2003	Defender	U15
19	8	2003	Defender	U15
27	9	2003	Defender	U15
11	12	2003	Defender	U15
15	1	2001	Forward	U15
20	1	2003	Forward	U15
9	2	2003	Forward	U15
17	2	2003	Forward	U15
15	3	2003	Forward	U15
24	3	2001	Forward	U15
1	4	2003	Forward	U15
8	4	2001	Forward	U15
22	4	2002	Forward	U15
13	5	2003	Forward	U15
13	5	2002	Forward	U15
16	5	2002	Forward	U15
20	5	2003	Forward	U15

DAY	MONTH	YEAR	ROLE	CATEGORY
2	6	2003	Forward	U15
7	6	2001	Forward	U15
10	6	2003	Forward	U15
11	7	2003	Forward	U15
4	8	2003	Forward	U15
8	8	2002	Forward	U15
3	9	2003	Forward	U15
16	9	2001	Forward	U15
1	10	2001	Forward	U15
24	10	2003	Forward	U15
18	12	2003	Forward	U15
1	1	2002	Forward	U15
19	1	2003	Forward	U15
20	1	2002	Forward	U15
27	1	2002	Forward	U15
2	2	2002	Forward	U15
6	2	2003	Forward	U15
11	2	2002	Forward	U15
28	2	2003	Forward	U15
3	3	2003	Forward	U15
8	3	2002	Forward	U15
18	3	2002	Forward	U15
30	3	2003	Forward	U15
7	4	2002	Forward	U15
22	4	2003	Forward	U15
9	5	2003	Forward	U15
9	5	2002	Forward	U15
14	5	2003	Forward	U15
25	5	2003	Forward	U15
28	5	2003	Forward	U15
3	6	2002	Forward	U15
11	6	2003	Forward	U15
24	6	2002	Forward	U15
23	7	2003	Forward	U15
30	7	2002	Forward	U15
28	8	2003	Forward	U15
23	9	2002	Forward	U15
20	10	2002	Forward	U15
23	10	2002	Forward	U15
23	1	2002	Goalkeeper	U15
25	1	2001	Goalkeeper	U15
7	3	2003	Goalkeeper	U15
9	3	2001	Goalkeeper	U15
24	3	2003	Goalkeeper	U15
31	3	2001	Goalkeeper	U15
7	4	2003	Goalkeeper	U15
11	5	2003	Goalkeeper	U15
14	7	2003	Goalkeeper	U15
21	7	2003	Goalkeeper	U15
4	1	2002	Goalkeeper	U15
4	1	2003	Goalkeeper	U15
8	1	2002	Goalkeeper	U15
16	1	2002	Goalkeeper	U15
18	1	2002	Goalkeeper	U15
6	2	2003	Goalkeeper	U15
17	2	2002	Goalkeeper	U15
22	3	2003	Goalkeeper	U15
28	3	2003	Goalkeeper	U15
22	4	2003	Goalkeeper	U15
26	4	2003	Goalkeeper	U15
24	7	2003	Goalkeeper	U15
14	9	2003	Goalkeeper	U15
4	1	2003	Midfielder	U15
5	1	2002	Midfielder	U15
11	1	2001	Midfielder	U15
16	1	2003	Midfielder	U15
27	1	2003	Midfielder	U15
5	2	2002	Midfielder	U15
6	2	2003	Midfielder	U15
9	2	2003	Midfielder	U15
10	2	2003	Midfielder	U15
11	2	2002	Midfielder	U15
12	2	2003	Midfielder	U15
13	2	2003	Midfielder	U15
16	2	2002	Midfielder	U15
18	2	2003	Midfielder	U15
8	3	2003	Midfielder	U15
9	3	2003	Midfielder	U15
26	3	2003	Midfielder	U15
4	4	2003	Midfielder	U15
15	4	2003	Midfielder	U15
3	5	2001	Midfielder	U15

DAY	MONTH	YEAR	ROLE	CATEGORY
4	5	2002	Midfielder	U15
4	5	2003	Midfielder	U15
9	5	2001	Midfielder	U15
9	5	2001	Midfielder	U15
12	5	2003	Midfielder	U15
29	5	2003	Midfielder	U15
30	5	2003	Midfielder	U15
22	6	2001	Midfielder	U15
28	6	2003	Midfielder	U15
30	6	2003	Midfielder	U15
4	7	2002	Midfielder	U15
29	7	2003	Midfielder	U15
1	8	2002	Midfielder	U15
8	8	2003	Midfielder	U15
19	8	2003	Midfielder	U15
17	9	2002	Midfielder	U15
17	9	2003	Midfielder	U15
23	9	2003	Midfielder	U15
23	9	2001	Midfielder	U15
30	9	2002	Midfielder	U15
14	12	2001	Midfielder	U15
19	12	2002	Midfielder	U15
2	1	2002	Midfielder	U15
3	1	2003	Midfielder	U15
11	1	2002	Midfielder	U15
14	1	2003	Midfielder	U15
17	1	2002	Midfielder	U15
17	1	2002	Midfielder	U15
24	1	2002	Midfielder	U15
26	1	2003	Midfielder	U15
30	1	2002	Midfielder	U15
1	2	2003	Midfielder	U15
18	2	2003	Midfielder	U15
21	2	2003	Midfielder	U15
23	2	2002	Midfielder	U15
4	3	2003	Midfielder	U15
20	3	2003	Midfielder	U15
25	3	2003	Midfielder	U15
25	3	2003	Midfielder	U15
26	3	2002	Midfielder	U15
28	3	2003	Midfielder	U15
1	4	2003	Midfielder	U15
6	4	2002	Midfielder	U15
27	4	2002	Midfielder	U15
27	4	2003	Midfielder	U15
4	6	2003	Midfielder	U15
24	6	2002	Midfielder	U15
2	7	2002	Midfielder	U15
23	8	2002	Midfielder	U15
30	8	2003	Midfielder	U15
13	9	2002	Midfielder	U15
21	10	2002	Midfielder	U15
6	1	2002	NaN	U15
13	1	2002	NaN	U15
23	1	2002	NaN	U15
3	2	2002	NaN	U15
4	2	2002	NaN	U15
6	2	2002	NaN	U15
12	2	2002	NaN	U15
7	3	2002	NaN	U15
1	4	2002	NaN	U15
6	4	2002	NaN	U15
9	4	2002	NaN	U15
12	4	2002	NaN	U15
19	4	2002	NaN	U15
16	5	2002	NaN	U15
19	5	2002	NaN	U15
23	5	2002	NaN	U15
7	6	2002	NaN	U15
21	6	2002	NaN	U15
28	6	2002	NaN	U15
25	7	2002	NaN	U15
31	7	2002	NaN	U15
10	9	2002	NaN	U15
11	11	2002	NaN	U15
23	11	2002	NaN	U15
2	1	2002	NaN	U15
3	1	2002	NaN	U15
18	1	2002	NaN	U15
4	2	2002	NaN	U15
7	2	2002	NaN	U15
14	2	2002	NaN	U15

DAY	MONTH	YEAR	ROLE	CATEGORY
16	2	2002	NaN	U15
22	2	2002	NaN	U15
23	2	2002	NaN	U15
12	3	2002	NaN	U15
15	3	2002	NaN	U15
29	3	2002	NaN	U15
1	4	2002	NaN	U15
22	4	2002	NaN	U15
28	4	2002	NaN	U15
4	5	2002	NaN	U15
30	5	2002	NaN	U15
19	7	2002	NaN	U15
21	7	2002	NaN	U15
3	8	2002	NaN	U15
4	8	2002	NaN	U15
7	8	2002	NaN	U15
14	8	2002	NaN	U15
2	9	2002	NaN	U15
24	10	2002	NaN	U15
12	1	2001	Defender	U16
16	1	2001	Defender	U16
19	1	2000	Defender	U16
19	1	2002	Defender	U16
21	1	2001	Defender	U16
22	1	2002	Defender	U16
1	2	2002	Defender	U16
1	2	2002	Defender	U16
4	2	2000	Defender	U16
11	2	2001	Defender	U16
28	2	2000	Defender	U16
7	3	2002	Defender	U16
16	3	2000	Defender	U16
1	4	2000	Defender	U16
9	4	2001	Defender	U16
9	4	2001	Defender	U16
20	4	2002	Defender	U16
21	4	2001	Defender	U16
29	4	2002	Defender	U16
19	5	2002	Defender	U16
29	5	2000	Defender	U16
23	6	2002	Defender	U16
23	6	2001	Defender	U16
24	6	2002	Defender	U16
11	7	2002	Defender	U16
13	7	2002	Defender	U16
25	7	2001	Defender	U16
2	8	2000	Defender	U16
19	8	2002	Defender	U16
20	8	2000	Defender	U16
28	8	2002	Defender	U16
10	10	2002	Defender	U16
15	10	2002	Defender	U16
30	12	2002	Defender	U16
1	1	2001	Defender	U16
1	1	2002	Defender	U16
2	1	2002	Defender	U16
4	1	2002	Defender	U16
5	1	2002	Defender	U16
7	1	2002	Defender	U16
10	1	2002	Defender	U16
10	1	2002	Defender	U16
11	1	2000	Defender	U16
11	1	2002	Defender	U16
15	1	2001	Defender	U16
19	1	2002	Defender	U16
22	1	2000	Defender	U16
23	1	2002	Defender	U16
23	1	2002	Defender	U16
23	1	2002	Defender	U16
24	1	2002	Defender	U16
26	1	2002	Defender	U16
26	1	2002	Defender	U16
2	2	2001	Defender	U16
2	2	2002	Defender	U16
6	2	2002	Defender	U16
10	2	2001	Defender	U16
17	2	2001	Defender	U16
23	2	2001	Defender	U16
29	2	2000	Defender	U16
1	3	2000	Defender	U16
5	3	2002	Defender	U16
6	3	2001	Defender	U16

DAY	MONTH	YEAR	ROLE	CATEGORY
7	3	2001	Defender	U16
8	3	2002	Defender	U16
11	3	2002	Defender	U16
11	3	2000	Defender	U16
13	3	2001	Defender	U16
19	3	2000	Defender	U16
24	3	2002	Defender	U16
26	3	2001	Defender	U16
28	3	2002	Defender	U16
4	4	2000	Defender	U16
6	4	2002	Defender	U16
7	4	2000	Defender	U16
17	4	2002	Defender	U16
18	4	2002	Defender	U16
19	4	2002	Defender	U16
22	4	2002	Defender	U16
30	4	2001	Defender	U16
4	5	2000	Defender	U16
7	5	2002	Defender	U16
18	5	2002	Defender	U16
22	5	2002	Defender	U16
2	6	2002	Defender	U16
3	6	2002	Defender	U16
12	6	2002	Defender	U16
18	6	2001	Defender	U16
20	6	2001	Defender	U16
9	7	2001	Defender	U16
11	7	2002	Defender	U16
16	7	2001	Defender	U16
27	7	2001	Defender	U16
30	7	2002	Defender	U16
4	8	2002	Defender	U16
7	8	2002	Defender	U16
9	8	2001	Defender	U16
10	8	2001	Defender	U16
16	8	2001	Defender	U16
6	9	2002	Defender	U16
10	9	2001	Defender	U16
30	9	2002	Defender	U16
16	11	2001	Defender	U16
27	11	2001	Defender	U16
1	1	2000	Forward	U16
24	1	2000	Forward	U16
30	1	2002	Forward	U16
7	2	2002	Forward	U16
10	2	2000	Forward	U16
11	2	2002	Forward	U16
19	3	2000	Forward	U16
26	3	2000	Forward	U16
16	4	2000	Forward	U16
22	4	2002	Forward	U16
1	5	2000	Forward	U16
21	5	2001	Forward	U16
3	6	2001	Forward	U16
21	6	2001	Forward	U16
14	7	2000	Forward	U16
1	8	2002	Forward	U16
3	8	2002	Forward	U16
8	8	2002	Forward	U16
27	9	2001	Forward	U16
25	10	2002	Forward	U16
1	1	2002	Forward	U16
1	1	2001	Forward	U16
1	1	2002	Forward	U16
1	1	2002	Forward	U16
1	1	2002	Forward	U16
2	1	2001	Forward	U16
2	1	2000	Forward	U16
8	1	2002	Forward	U16
8	1	2001	Forward	U16
8	1	2000	Forward	U16
10	1	2002	Forward	U16
12	1	2002	Forward	U16
14	1	2001	Forward	U16
18	1	2002	Forward	U16
22	1	2002	Forward	U16
25	1	2001	Forward	U16
26	1	2001	Forward	U16
27	1	2002	Forward	U16
27	1	2000	Forward	U16
29	1	2002	Forward	U16
30	1	2001	Forward	U16

DAY	MONTH	YEAR	ROLE	CATEGORY
1	2	2001	Forward	U16
1	2	2002	Forward	U16
5	2	2002	Forward	U16
5	2	2002	Forward	U16
5	2	2000	Forward	U16
7	2	2001	Forward	U16
16	2	2002	Forward	U16
23	2	2002	Forward	U16
3	3	2001	Forward	U16
6	3	2001	Forward	U16
12	3	2002	Forward	U16
17	3	2001	Forward	U16
26	3	2002	Forward	U16
27	3	2002	Forward	U16
6	4	2000	Forward	U16
14	4	2001	Forward	U16
30	4	2000	Forward	U16
3	5	2002	Forward	U16
7	5	2001	Forward	U16
16	5	2002	Forward	U16
19	5	2001	Forward	U16
23	5	2001	Forward	U16
29	5	2002	Forward	U16
9	6	2002	Forward	U16
14	6	2000	Forward	U16
18	6	2002	Forward	U16
29	6	2002	Forward	U16
2	7	2002	Forward	U16
7	7	2002	Forward	U16
14	7	2000	Forward	U16
17	7	2002	Forward	U16
19	7	2001	Forward	U16
19	7	2002	Forward	U16
21	7	2002	Forward	U16
27	7	2001	Forward	U16
1	8	2002	Forward	U16
22	8	2002	Forward	U16
27	8	2002	Forward	U16
19	9	2002	Forward	U16
21	9	2001	Forward	U16
27	9	2001	Forward	U16
27	9	2002	Forward	U16
7	10	2000	Forward	U16
11	10	2001	Forward	U16
26	10	2002	Forward	U16
30	10	2001	Forward	U16
30	10	2003	Forward	U16
30	10	2002	Forward	U16
21	11	2002	Forward	U16
12	1	2000	Goalkeeper	U16
16	1	2001	Goalkeeper	U16
26	1	2002	Goalkeeper	U16
23	2	2000	Goalkeeper	U16
28	2	2002	Goalkeeper	U16
16	3	2002	Goalkeeper	U16
6	1	2002	Goalkeeper	U16
16	1	2002	Goalkeeper	U16
26	1	2002	Goalkeeper	U16
28	1	2000	Goalkeeper	U16
3	2	2002	Goalkeeper	U16
4	2	2001	Goalkeeper	U16
17	2	2001	Goalkeeper	U16
17	2	2002	Goalkeeper	U16
20	2	2002	Goalkeeper	U16
20	2	2002	Goalkeeper	U16
21	2	2001	Goalkeeper	U16
18	3	2002	Goalkeeper	U16
28	3	2001	Goalkeeper	U16
1	4	2002	Goalkeeper	U16
9	4	2002	Goalkeeper	U16
19	4	2002	Goalkeeper	U16
2	5	2001	Goalkeeper	U16
11	5	2001	Goalkeeper	U16
17	5	2002	Goalkeeper	U16
17	5	2002	Goalkeeper	U16
25	5	2002	Goalkeeper	U16
8	7	2001	Goalkeeper	U16
6	8	2002	Goalkeeper	U16
7	8	2001	Goalkeeper	U16
8	8	2001	Goalkeeper	U16
17	8	2002	Goalkeeper	U16
25	8	2001	Goalkeeper	U16

DAY	MONTH	YEAR	ROLE	CATEGORY
20	10	2002	Goalkeeper	U16
11	12	2002	Goalkeeper	U16
1	1	2001	Midfielder	U16
4	1	2002	Midfielder	U16
7	1	2002	Midfielder	U16
8	1	2002	Midfielder	U16
12	1	2002	Midfielder	U16
19	1	2002	Midfielder	U16
20	1	2000	Midfielder	U16
26	1	2000	Midfielder	U16
29	1	2001	Midfielder	U16
1	2	2001	Midfielder	U16
5	2	2001	Midfielder	U16
6	2	2001	Midfielder	U16
9	2	2002	Midfielder	U16
18	2	2001	Midfielder	U16
20	2	2000	Midfielder	U16
24	2	2002	Midfielder	U16
26	2	2001	Midfielder	U16
5	3	2002	Midfielder	U16
27	3	2001	Midfielder	U16
28	3	2000	Midfielder	U16
5	4	2002	Midfielder	U16
19	4	2002	Midfielder	U16
30	4	2002	Midfielder	U16
14	5	2002	Midfielder	U16
31	5	2002	Midfielder	U16
14	6	2002	Midfielder	U16
21	6	2002	Midfielder	U16
4	7	2002	Midfielder	U16
19	7	2001	Midfielder	U16
20	7	2001	Midfielder	U16
7	8	2000	Midfielder	U16
20	8	2000	Midfielder	U16
27	8	2002	Midfielder	U16
18	10	2002	Midfielder	U16
1	1	2000	Midfielder	U16
1	1	2001	Midfielder	U16
1	1	2002	Midfielder	U16
1	1	2002	Midfielder	U16
1	1	2002	Midfielder	U16
4	1	2001	Midfielder	U16
4	1	2002	Midfielder	U16
4	1	2002	Midfielder	U16
8	1	2000	Midfielder	U16
11	1	2002	Midfielder	U16
16	1	2001	Midfielder	U16
18	1	2001	Midfielder	U16
18	1	2002	Midfielder	U16
19	1	2000	Midfielder	U16
19	1	2002	Midfielder	U16
25	1	2002	Midfielder	U16
27	1	2001	Midfielder	U16
28	1	2002	Midfielder	U16
1	2	2002	Midfielder	U16
4	2	2002	Midfielder	U16
7	2	2000	Midfielder	U16
18	2	2001	Midfielder	U16
18	2	2002	Midfielder	U16
24	2	2001	Midfielder	U16
8	3	2002	Midfielder	U16
9	3	2002	Midfielder	U16
11	3	2002	Midfielder	U16
13	3	2002	Midfielder	U16
14	3	2001	Midfielder	U16
17	3	2002	Midfielder	U16
17	3	2002	Midfielder	U16
23	3	2001	Midfielder	U16
23	3	2002	Midfielder	U16
24	3	2001	Midfielder	U16
8	4	2001	Midfielder	U16
11	4	2002	Midfielder	U16
11	4	2000	Midfielder	U16
12	4	2002	Midfielder	U16
14	4	2002	Midfielder	U16
16	4	2002	Midfielder	U16
19	4	2002	Midfielder	U16
21	4	2001	Midfielder	U16
22	4	2002	Midfielder	U16
22	4	2002	Midfielder	U16
24	4	2001	Midfielder	U16
25	4	2000	Midfielder	U16

DAY	MONTH	YEAR	ROLE	CATEGORY
28	4	2001	Midfielder	U16
29	4	2002	Midfielder	U16
7	5	2001	Midfielder	U16
8	5	2002	Midfielder	U16
13	5	2001	Midfielder	U16
14	5	2001	Midfielder	U16
14	5	2002	Midfielder	U16
16	5	2002	Midfielder	U16
26	5	2002	Midfielder	U16
1	6	2000	Midfielder	U16
10	6	2001	Midfielder	U16
16	6	2001	Midfielder	U16
17	6	2002	Midfielder	U16
24	6	2002	Midfielder	U16
30	6	2000	Midfielder	U16
1	7	2002	Midfielder	U16
12	7	2001	Midfielder	U16
15	7	2001	Midfielder	U16
25	7	2001	Midfielder	U16
25	7	2001	Midfielder	U16
15	8	2002	Midfielder	U16
10	9	2001	Midfielder	U16
15	9	2000	Midfielder	U16
8	10	2001	Midfielder	U16
22	10	2002	Midfielder	U16
25	10	2002	Midfielder	U16
29	11	2002	Midfielder	U16
1	12	2001	Midfielder	U16
4	12	2002	Midfielder	U16
1	1	2001	NaN	U16
4	1	2001	NaN	U16
10	1	2001	NaN	U16
14	1	2001	NaN	U16
15	1	2001	NaN	U16
18	1	2001	NaN	U16
31	1	2001	NaN	U16
9	2	2001	NaN	U16
12	2	2001	NaN	U16
14	2	2001	NaN	U16
28	2	2001	NaN	U16
7	3	2001	NaN	U16
15	3	2001	NaN	U16
1	4	2001	NaN	U16
17	4	2001	NaN	U16
19	4	2001	NaN	U16
1	5	2001	NaN	U16
10	5	2001	NaN	U16
25	5	2001	NaN	U16
1	6	2002	NaN	U16
28	6	2001	NaN	U16
26	9	2001	NaN	U16
18	10	2001	NaN	U16
16	11	2001	NaN	U16
5	3	2000	Defender	U17
8	1	2000	Defender	U17
16	3	2000	Defender	U17
31	1	2000	Defender	U17
12	1	2001	Defender	U17
28	1	2001	Defender	U17
5	10	2001	Defender	U17
3	11	2001	Defender	U17
20	6	2001	Defender	U17
13	7	2001	Defender	U17
6	3	2001	Defender	U17
27	3	2001	Defender	U17
7	3	2001	Defender	U17
7	3	2001	Defender	U17
26	10	2000	Defender	U17
25	9	2000	Defender	U17
12	4	2000	Defender	U17
11	4	2001	Defender	U17
24	3	2001	Defender	U17
12	12	2001	Defender	U17
16	5	2001	Defender	U17
18	2	2001	Defender	U17
15	1	2001	Defender	U17
18	4	2001	Defender	U17
11	5	2001	Defender	U17
12	9	2001	Defender	U17
12	9	2001	Defender	U17
12	2	2001	Defender	U17
10	1	2001	Defender	U17

DAY	MONTH	YEAR	ROLE	CATEGORY
15	3	2001	Defender	U17
22	1	2001	Defender	U17
1	9	2001	Defender	U17
27	10	2001	Defender	U17
4	8	2001	Defender	U17
3	7	2001	Defender	U17
24	1	2000	Defender	U17
21	5	2000	Defender	U17
21	4	2001	Defender	U17
12	1	2000	Defender	U17
15	5	2001	Defender	U17
3	5	2000	Defender	U17
11	10	2000	Defender	U17
14	9	2000	Defender	U17
9	4	2001	Defender	U17
13	1	2000	Defender	U17
1	1	2001	Defender	U17
25	12	2001	Defender	U17
1	1	2001	Defender	U17
6	6	2001	Defender	U17
4	2	2001	Defender	U17
6	2	2001	Defender	U17
4	9	2001	Defender	U17
28	3	2001	Defender	U17
11	6	2001	Defender	U17
28	6	2001	Defender	U17
1	1	2001	Defender	U17
29	5	2001	Defender	U17
25	3	2001	Defender	U17
2	5	2001	Defender	U17
10	10	2001	Defender	U17
2	2	2001	Defender	U17
8	5	2001	Defender	U17
4	4	2001	Defender	U17
15	7	2002	Defender	U17
25	7	2001	Defender	U17
1	1	2001	Defender	U17
17	1	2001	Defender	U17
4	2	2001	Defender	U17
20	2	2002	Defender	U17
25	5	2001	Defender	U17
12	2	2001	Defender	U17
17	1	2001	Defender	U17
23	7	2002	Defender	U17
24	5	2001	Defender	U17
21	3	2001	Defender	U17
27	1	2001	Defender	U17
2	2	2001	Defender	U17
28	4	2001	Defender	U17
9	5	2001	Defender	U17
29	9	2001	Defender	U17
15	5	2001	Defender	U17
28	2	2000	Defender	U17
25	10	2001	Defender	U17
8	5	2001	Defender	U17
1	1	2001	Defender	U17
20	7	2001	Defender	U17
1	1	2001	Defender	U17
11	9	2001	Defender	U17
6	8	2001	Defender	U17
15	5	2001	Defender	U17
31	5	2001	Defender	U17
21	2	2001	Defender	U17
1	9	2001	Defender	U17
10	7	2001	Defender	U17
18	7	2001	Defender	U17
1	3	2001	Defender	U17
24	5	2001	Defender	U17
21	3	2001	Defender	U17
27	1	2001	Defender	U17
2	2	2001	Defender	U17
28	4	2001	Defender	U17
9	5	2001	Defender	U17
29	9	2001	Defender	U17
15	5	2001	Defender	U17
28	2	2000	Defender	U17
4	9	2001	Defender	U17
23	1	2001	Defender	U17
4	3	2001	Defender	U17
10	8	2001	Defender	U17
5	3	2002	Defender	U17
27	3	2001	Defender	U17

DAY	MONTH	YEAR	ROLE	CATEGORY
6	3	2001	Defender	U17
10	1	2001	Defender	U17
1	1	2001	Defender	U17
7	3	2001	Defender	U17
18	10	2001	Defender	U17
9	4	2002	Defender	U17
19	5	2002	Defender	U17
10	4	2001	Defender	U17
22	6	2001	Defender	U17
19	6	2001	Defender	U17
9	6	2001	Defender	U17
3	3	2001	Defender	U17
20	11	2001	Defender	U17
15	2	2001	Defender	U17
2	11	2000	Forward	U17
28	3	2000	Forward	U17
9	10	2000	Forward	U17
25	2	2000	Forward	U17
29	1	2000	Forward	U17
1	2	2000	Forward	U17
11	10	2000	Forward	U17
6	1	2000	Forward	U17
4	1	2001	Forward	U17
15	2	2001	Forward	U17
17	8	2001	Forward	U17
5	10	2001	Forward	U17
10	7	2001	Forward	U17
27	1	2001	Forward	U17
25	2	2001	Forward	U17
5	1	2001	Forward	U17
11	7	2002	Forward	U17
23	5	2000	Forward	U17
22	5	2000	Forward	U17
19	7	2000	Forward	U17
7	1	2000	Forward	U17
2	7	2000	Forward	U17
25	5	2001	Forward	U17
8	2	2001	Forward	U17
4	12	2001	Forward	U17
23	7	2001	Forward	U17
12	3	2001	Forward	U17
26	5	2001	Forward	U17
22	10	2001	Forward	U17
10	6	2001	Forward	U17
22	5	2001	Forward	U17
26	3	2001	Forward	U17
2	2	2001	Forward	U17
6	3	2001	Forward	U17
17	7	2001	Forward	U17
3	7	2001	Forward	U17
15	2	2001	Forward	U17
5	8	2001	Forward	U17
24	5	2001	Forward	U17
9	1	2001	Forward	U17
21	5	2001	Forward	U17
11	7	2001	Forward	U17
7	4	2000	Forward	U17
26	1	2000	Forward	U17
14	8	2000	Forward	U17
7	4	2000	Forward	U17
26	2	2001	Forward	U17
18	6	2001	Forward	U17
6	7	2001	Forward	U17
1	1	2001	Forward	U17
4	1	2001	Forward	U17
1	1	2001	Forward	U17
21	3	2002	Forward	U17
14	10	2001	Forward	U17
29	3	2001	Forward	U17
25	2	2001	Forward	U17
24	1	2001	Forward	U17
22	8	2001	Forward	U17
2	7	2002	Forward	U17
19	10	2001	Forward	U17
6	2	2001	Forward	U17
12	3	2001	Forward	U17
23	9	2001	Forward	U17
10	3	2001	Forward	U17
25	1	2001	Forward	U17
23	1	2001	Forward	U17
24	3	2001	Forward	U17
12	3	2001	Forward	U17

DAY	MONTH	YEAR	ROLE	CATEGORY
13	4	2001	Forward	U17
22	1	2001	Forward	U17
10	4	2001	Forward	U17
8	8	2001	Forward	U17
16	9	2001	Forward	U17
17	3	2001	Forward	U17
19	5	2001	Forward	U17
4	1	2001	Forward	U17
6	12	2001	Forward	U17
9	9	2001	Forward	U17
23	9	2001	Forward	U17
10	3	2001	Forward	U17
25	1	2001	Forward	U17
23	1	2001	Forward	U17
24	3	2001	Forward	U17
29	7	2001	Forward	U17
7	8	2002	Forward	U17
3	5	2001	Forward	U17
22	1	2001	Forward	U17
2	4	2001	Forward	U17
8	4	2001	Forward	U17
16	11	2001	Forward	U17
15	1	2001	Forward	U17
15	3	2001	Forward	U17
1	6	2002	Forward	U17
10	5	2001	Forward	U17
20	12	2001	Forward	U17
6	5	2001	Forward	U17
6	6	2001	Forward	U17
19	6	2001	Forward	U17
24	2	2001	Forward	U17
28	2	2001	Forward	U17
8	5	2001	Forward	U17
31	1	2000	Goalkeeper	U17
28	3	2001	Goalkeeper	U17
21	8	2001	Goalkeeper	U17
4	2	2001	Goalkeeper	U17
26	4	2000	Goalkeeper	U17
16	12	2001	Goalkeeper	U17
21	8	2001	Goalkeeper	U17
23	8	2001	Goalkeeper	U17
14	7	2001	Goalkeeper	U17
16	3	2001	Goalkeeper	U17
31	3	2001	Goalkeeper	U17
6	8	2001	Goalkeeper	U17
2	8	2001	Goalkeeper	U17
27	7	2001	Goalkeeper	U17
2	1	2000	Goalkeeper	U17
6	7	2000	Goalkeeper	U17
20	7	2000	Goalkeeper	U17
1	1	2001	Goalkeeper	U17
14	8	2001	Goalkeeper	U17
8	6	2001	Goalkeeper	U17
24	1	2002	Goalkeeper	U17
4	2	2001	Goalkeeper	U17
22	2	2001	Goalkeeper	U17
19	2	2001	Goalkeeper	U17
1	1	2001	Goalkeeper	U17
5	4	2001	Goalkeeper	U17
31	3	2001	Goalkeeper	U17
5	4	2001	Goalkeeper	U17
16	1	2001	Goalkeeper	U17
16	3	2001	Goalkeeper	U17
6	3	2001	Goalkeeper	U17
11	5	2001	Goalkeeper	U17
5	9	2001	Goalkeeper	U17
31	3	2001	Goalkeeper	U17
5	4	2001	Goalkeeper	U17
16	1	2001	Goalkeeper	U17
28	6	2001	Goalkeeper	U17
14	4	2001	Goalkeeper	U17
26	8	2002	Goalkeeper	U17
28	6	2001	Goalkeeper	U17
9	2	2001	Goalkeeper	U17
28	2	2001	Goalkeeper	U17
5	1	2001	Goalkeeper	U17
18	4	2001	Goalkeeper	U17
2	10	2000	Midfielder	U17
20	1	2000	Midfielder	U17
2	12	2000	Midfielder	U17
3	6	2000	Midfielder	U17
22	1	2000	Midfielder	U17

DAY	MONTH	YEAR	ROLE	CATEGORY
7	3	2000	Midfielder	U17
17	4	2000	Midfielder	U17
4	5	2000	Midfielder	U17
25	1	2001	Midfielder	U17
20	2	2001	Midfielder	U17
18	2	2001	Midfielder	U17
16	6	2001	Midfielder	U17
25	5	2001	Midfielder	U17
21	9	2001	Midfielder	U17
23	2	2001	Midfielder	U17
23	3	2001	Midfielder	U17
25	11	2000	Midfielder	U17
8	4	2000	Midfielder	U17
16	4	2000	Midfielder	U17
27	2	2000	Midfielder	U17
3	3	2000	Midfielder	U17
2	6	2000	Midfielder	U17
30	1	2001	Midfielder	U17
19	9	2001	Midfielder	U17
9	11	2001	Midfielder	U17
22	5	2001	Midfielder	U17
22	8	2001	Midfielder	U17
6	7	2001	Midfielder	U17
14	8	2001	Midfielder	U17
5	3	2001	Midfielder	U17
19	2	2001	Midfielder	U17
13	2	2001	Midfielder	U17
31	3	2001	Midfielder	U17
16	7	2001	Midfielder	U17
17	5	2001	Midfielder	U17
8	1	2001	Midfielder	U17
30	4	2001	Midfielder	U17
8	11	2001	Midfielder	U17
14	8	2001	Midfielder	U17
4	12	2001	Midfielder	U17
23	3	2001	Midfielder	U17
11	4	2001	Midfielder	U17
5	4	2001	Midfielder	U17
21	12	2001	Midfielder	U17
19	2	2000	Midfielder	U17
12	2	2000	Midfielder	U17
31	1	2001	Midfielder	U17
13	2	2000	Midfielder	U17
28	8	2000	Midfielder	U17
9	3	2000	Midfielder	U17
24	11	2001	Midfielder	U17
18	2	2001	Midfielder	U17
1	2	2001	Midfielder	U17
20	5	2001	Midfielder	U17
17	5	2001	Midfielder	U17
17	3	2001	Midfielder	U17
21	4	2001	Midfielder	U17
21	8	2001	Midfielder	U17
24	4	2001	Midfielder	U17
23	10	2001	Midfielder	U17
24	4	2001	Midfielder	U17
3	5	2001	Midfielder	U17
9	10	2001	Midfielder	U17
9	4	2001	Midfielder	U17
28	11	2001	Midfielder	U17
3	4	2001	Midfielder	U17
14	1	2001	Midfielder	U17
1	1	2001	Midfielder	U17
1	1	2002	Midfielder	U17
11	1	2001	Midfielder	U17
5	4	2001	Midfielder	U17
7	1	2002	Midfielder	U17
1	1	2001	Midfielder	U17
4	3	2001	Midfielder	U17
5	5	2001	Midfielder	U17
1	1	2001	Midfielder	U17
27	4	2001	Midfielder	U17
4	12	2001	Midfielder	U17
9	5	2001	Midfielder	U17
7	6	2001	Midfielder	U17
14	12	2001	Midfielder	U17
2	4	2001	Midfielder	U17
28	10	2001	Midfielder	U17
26	11	2001	Midfielder	U17
10	3	2002	Midfielder	U17
1	1	2001	Midfielder	U17
15	4	2001	Midfielder	U17

DAY	MONTH	YEAR	ROLE	CATEGORY
8	4	2001	Midfielder	U17
1	1	2001	Midfielder	U17
30	4	2001	Midfielder	U17
12	3	2001	Midfielder	U17
27	3	2001	Midfielder	U17
12	6	2001	Midfielder	U17
3	7	2001	Midfielder	U17
20	6	2001	Midfielder	U17
31	1	2001	Midfielder	U17
22	1	2001	Midfielder	U17
5	10	2001	Midfielder	U17
26	3	2001	Midfielder	U17
21	12	2001	Midfielder	U17
18	3	2001	Midfielder	U17
27	8	2001	Midfielder	U17
11	1	2001	Midfielder	U17
27	4	2001	Midfielder	U17
4	12	2001	Midfielder	U17
9	5	2001	Midfielder	U17
7	6	2001	Midfielder	U17
14	12	2001	Midfielder	U17
2	4	2001	Midfielder	U17
10	9	2001	Midfielder	U17
7	9	2001	Midfielder	U17
6	11	2001	Midfielder	U17
23	3	2001	Midfielder	U17
19	3	2002	Midfielder	U17
1	5	2001	Midfielder	U17
5	10	2001	Midfielder	U17
31	1	2001	Midfielder	U17
1	4	2001	Midfielder	U17
4	1	2001	Midfielder	U17
17	4	2001	Midfielder	U17
26	9	2001	Midfielder	U17
19	4	2001	Midfielder	U17
25	5	2001	Midfielder	U17
18	1	2001	Midfielder	U17
12	2	2001	Midfielder	U17
5	5	2001	Midfielder	U17
24	3	2001	Midfielder	U17
10	8	2001	Midfielder	U17
12	7	2001	Midfielder	U17
21	7	2001	Midfielder	U17
26	2	2001	Midfielder	U17
17	5	2001	Midfielder	U17
4	8	2000	NaN	U17
4	1	1999	Defender	Primavara
4	1	1998	Defender	Primavara
6	1	2001	Defender	Primavara
8	1	1998	Defender	Primavara
10	1	1998	Defender	Primavara
10	1	2000	Defender	Primavara
13	1	1998	Defender	Primavara
14	1	1999	Defender	Primavara
14	1	1999	Defender	Primavara
17	1	1999	Defender	Primavara
18	1	1999	Defender	Primavara
19	1	2000	Defender	Primavara
20	1	1999	Defender	Primavara
21	1	1999	Defender	Primavara
24	1	2000	Defender	Primavara
25	1	1999	Defender	Primavara
28	1	2000	Defender	Primavara
1	2	2000	Defender	Primavara
2	2	1999	Defender	Primavara
3	2	1998	Defender	Primavara
4	2	1999	Defender	Primavara
5	2	1998	Defender	Primavara
6	2	1999	Defender	Primavara
7	2	1999	Defender	Primavara
8	2	1999	Defender	Primavara
8	2	2000	Defender	Primavara
9	2	1999	Defender	Primavara
11	2	2000	Defender	Primavara
11	2	2000	Defender	Primavara
11	2	1999	Defender	Primavara
17	2	1999	Defender	Primavara
18	2	1999	Defender	Primavara
21	2	2000	Defender	Primavara
24	2	2000	Defender	Primavara
28	2	2000	Defender	Primavara
1	3	1999	Defender	Primavara

DAY	MONTH	YEAR	ROLE	CATEGORY
2	3	1999	Defender	Primavara
3	3	1999	Defender	Primavara
3	3	1999	Defender	Primavara
4	3	1999	Defender	Primavara
5	3	1999	Defender	Primavara
6	3	2000	Defender	Primavara
7	3	2000	Defender	Primavara
7	3	1999	Defender	Primavara
11	3	2000	Defender	Primavara
16	3	2000	Defender	Primavara
20	3	1999	Defender	Primavara
20	3	1999	Defender	Primavara
20	3	1999	Defender	Primavara
20	3	1999	Defender	Primavara
24	3	2000	Defender	Primavara
26	3	2000	Defender	Primavara
29	3	2000	Defender	Primavara
30	3	1998	Defender	Primavara
7	4	2000	Defender	Primavara
7	4	1999	Defender	Primavara
8	4	1999	Defender	Primavara
9	4	2000	Defender	Primavara
12	4	2000	Defender	Primavara
12	4	2000	Defender	Primavara
16	4	1999	Defender	Primavara
18	4	2001	Defender	Primavara
19	4	2000	Defender	Primavara
20	4	1999	Defender	Primavara
20	4	1999	Defender	Primavara
23	4	1999	Defender	Primavara
24	4	1998	Defender	Primavara
1	5	1996	Defender	Primavara
1	5	2001	Defender	Primavara
3	5	1999	Defender	Primavara
6	5	2000	Defender	Primavara
7	5	1998	Defender	Primavara
17	5	2000	Defender	Primavara
22	5	1998	Defender	Primavara
23	5	2001	Defender	Primavara
25	5	1998	Defender	Primavara
27	5	1999	Defender	Primavara
28	5	1999	Defender	Primavara
29	5	2000	Defender	Primavara
4	6	1999	Defender	Primavara
6	6	1999	Defender	Primavara
7	6	2000	Defender	Primavara
14	6	2000	Defender	Primavara
17	6	1999	Defender	Primavara
18	6	1999	Defender	Primavara
21	6	2000	Defender	Primavara
24	6	1999	Defender	Primavara
25	6	1998	Defender	Primavara
26	6	1999	Defender	Primavara
28	6	1999	Defender	Primavara
29	6	1999	Defender	Primavara
30	6	1999	Defender	Primavara
7	7	1998	Defender	Primavara
16	7	1999	Defender	Primavara
23	7	1999	Defender	Primavara
26	7	2000	Defender	Primavara
29	7	1999	Defender	Primavara
2	8	2000	Defender	Primavara
9	8	1998	Defender	Primavara
11	8	1999	Defender	Primavara
13	8	2001	Defender	Primavara
16	8	2000	Defender	Primavara
20	8	1998	Defender	Primavara
23	8	1999	Defender	Primavara
27	8	1998	Defender	Primavara
2	9	2000	Defender	Primavara
10	9	1999	Defender	Primavara
14	9	1998	Defender	Primavara
21	9	1998	Defender	Primavara
22	9	1998	Defender	Primavara
29	9	1999	Defender	Primavara
29	9	1998	Defender	Primavara
29	9	1998	Defender	Primavara
1	10	1999	Defender	Primavara
19	10	2000	Defender	Primavara
26	10	2000	Defender	Primavara
30	10	2000	Defender	Primavara
6	11	1998	Defender	Primavara

DAY	MONTH	YEAR	ROLE	CATEGORY
9	11	1999	Defender	Primavara
10	11	1999	Defender	Primavara
10	11	2000	Defender	Primavara
11	11	2000	Defender	Primavara
14	12	1999	Defender	Primavara
19	12	1998	Defender	Primavara
30	12	1999	Defender	Primavara
1	1	1999	Forward	Primavara
2	1	1999	Forward	Primavara
2	1	1999	Forward	Primavara
2	1	2000	Forward	Primavara
4	1	1999	Forward	Primavara
5	1	2000	Forward	Primavara
6	1	1999	Forward	Primavara
6	1	1999	Forward	Primavara
6	1	1999	Forward	Primavara
6	1	2000	Forward	Primavara
7	1	2000	Forward	Primavara
7	1	1999	Forward	Primavara
10	1	1998	Forward	Primavara
19	1	1998	Forward	Primavara
24	1	2000	Forward	Primavara
25	1	1999	Forward	Primavara
27	1	2000	Forward	Primavara
1	2	2000	Forward	Primavara
1	2	1999	Forward	Primavara
2	2	2000	Forward	Primavara
6	2	2000	Forward	Primavara
8	2	1999	Forward	Primavara
13	2	1999	Forward	Primavara
17	2	2000	Forward	Primavara
18	2	2000	Forward	Primavara
19	2	2000	Forward	Primavara
20	2	1999	Forward	Primavara
20	2	1998	Forward	Primavara
23	2	1999	Forward	Primavara
23	2	2000	Forward	Primavara
27	2	1999	Forward	Primavara
1	3	1999	Forward	Primavara
3	3	2000	Forward	Primavara
9	3	2000	Forward	Primavara
10	3	1999	Forward	Primavara
11	3	1999	Forward	Primavara
13	3	1999	Forward	Primavara
14	3	1999	Forward	Primavara
14	3	2000	Forward	Primavara
17	3	2001	Forward	Primavara
18	3	1999	Forward	Primavara
21	3	1999	Forward	Primavara
22	3	2000	Forward	Primavara
24	3	2000	Forward	Primavara
27	3	1998	Forward	Primavara
30	3	2000	Forward	Primavara
31	3	1999	Forward	Primavara
10	4	2001	Forward	Primavara
15	4	1999	Forward	Primavara
17	4	2000	Forward	Primavara
22	4	2000	Forward	Primavara
23	4	2000	Forward	Primavara
25	4	1999	Forward	Primavara
28	4	1999	Forward	Primavara
29	4	1999	Forward	Primavara
30	4	2000	Forward	Primavara
4	5	1998	Forward	Primavara
5	5	2000	Forward	Primavara
10	5	1999	Forward	Primavara
19	5	2000	Forward	Primavara
22	5	1999	Forward	Primavara
22	5	1999	Forward	Primavara
26	5	1999	Forward	Primavara
28	5	1999	Forward	Primavara
31	5	1999	Forward	Primavara
3	6	1999	Forward	Primavara
6	6	1999	Forward	Primavara
10	6	2000	Forward	Primavara
11	6	2000	Forward	Primavara
14	6	2000	Forward	Primavara
18	6	2000	Forward	Primavara
19	6	2001	Forward	Primavara
27	6	2000	Forward	Primavara
30	6	1999	Forward	Primavara
30	6	1999	Forward	Primavara

DAY	MONTH	YEAR	ROLE	CATEGORY
3	7	1999	Forward	Primavara
8	7	1999	Forward	Primavara
13	7	2000	Forward	Primavara
17	7	1998	Forward	Primavara
17	7	1999	Forward	Primavara
19	7	2000	Forward	Primavara
30	7	1999	Forward	Primavara
6	8	1999	Forward	Primavara
12	8	2000	Forward	Primavara
16	8	1999	Forward	Primavara
20	8	1999	Forward	Primavara
21	8	1998	Forward	Primavara
3	9	1998	Forward	Primavara
4	9	1999	Forward	Primavara
1	10	2001	Forward	Primavara
9	10	2000	Forward	Primavara
12	10	1999	Forward	Primavara
18	10	1998	Forward	Primavara
10	11	2000	Forward	Primavara
12	11	1999	Forward	Primavara
14	11	1998	Forward	Primavara
21	11	1999	Forward	Primavara
4	12	1999	Forward	Primavara
5	12	1999	Forward	Primavara
8	12	2000	Forward	Primavara
9	12	1998	Forward	Primavara
3	1	1999	Goalkeeper	Primavara
4	1	1999	Goalkeeper	Primavara
4	1	1999	Goalkeeper	Primavara
8	1	1998	Goalkeeper	Primavara
9	1	2000	Goalkeeper	Primavara
12	1	2000	Goalkeeper	Primavara
20	1	2000	Goalkeeper	Primavara
22	1	2000	Goalkeeper	Primavara
28	1	1998	Goalkeeper	Primavara
29	1	1999	Goalkeeper	Primavara
31	1	2000	Goalkeeper	Primavara
21	2	1999	Goalkeeper	Primavara
23	2	2000	Goalkeeper	Primavara
25	2	2000	Goalkeeper	Primavara
16	3	2001	Goalkeeper	Primavara
17	3	2000	Goalkeeper	Primavara
19	3	2000	Goalkeeper	Primavara
20	3	1999	Goalkeeper	Primavara
22	3	2000	Goalkeeper	Primavara
27	3	1999	Goalkeeper	Primavara
28	3	2001	Goalkeeper	Primavara
31	3	2001	Goalkeeper	Primavara
26	4	2000	Goalkeeper	Primavara
28	4	1999	Goalkeeper	Primavara
25	5	1999	Goalkeeper	Primavara
5	6	1998	Goalkeeper	Primavara
21	6	2000	Goalkeeper	Primavara
30	6	2000	Goalkeeper	Primavara
1	7	2000	Goalkeeper	Primavara
1	7	2000	Goalkeeper	Primavara
3	7	2000	Goalkeeper	Primavara
6	7	1999	Goalkeeper	Primavara
11	7	2000	Goalkeeper	Primavara
11	7	1999	Goalkeeper	Primavara
14	7	2001	Goalkeeper	Primavara
27	7	2001	Goalkeeper	Primavara
29	7	1998	Goalkeeper	Primavara
8	8	1999	Goalkeeper	Primavara
10	8	1999	Goalkeeper	Primavara
23	9	1999	Goalkeeper	Primavara
7	10	1999	Goalkeeper	Primavara
30	10	1999	Goalkeeper	Primavara
11	11	1999	Goalkeeper	Primavara
12	11	1999	Goalkeeper	Primavara
25	11	1999	Goalkeeper	Primavara
1	1	1999	Midfielder	Primavara
1	1	1998	Midfielder	Primavara
2	1	2000	Midfielder	Primavara
3	1	1999	Midfielder	Primavara
5	1	2001	Midfielder	Primavara
6	1	2000	Midfielder	Primavara
7	1	1998	Midfielder	Primavara
7	1	2000	Midfielder	Primavara
7	1	1999	Midfielder	Primavara
9	1	2000	Midfielder	Primavara
10	1	1999	Midfielder	Primavara

DAY	MONTH	YEAR	ROLE	CATEGORY
15	1	2000	Midfielder	Primavara
17	1	1998	Midfielder	Primavara
20	1	2000	Midfielder	Primavara
20	1	1998	Midfielder	Primavara
21	1	2000	Midfielder	Primavara
21	1	2000	Midfielder	Primavara
23	1	2000	Midfielder	Primavara
28	1	1999	Midfielder	Primavara
30	1	1999	Midfielder	Primavara
30	1	1998	Midfielder	Primavara
30	1	2000	Midfielder	Primavara
30	1	1999	Midfielder	Primavara
30	1	2000	Midfielder	Primavara
1	2	1998	Midfielder	Primavara
4	2	1999	Midfielder	Primavara
4	2	2000	Midfielder	Primavara
5	2	1999	Midfielder	Primavara
7	2	1999	Midfielder	Primavara
7	2	2000	Midfielder	Primavara
8	2	1999	Midfielder	Primavara
10	2	1998	Midfielder	Primavara
10	2	1999	Midfielder	Primavara
12	2	2001	Midfielder	Primavara
12	2	2000	Midfielder	Primavara
18	2	1999	Midfielder	Primavara
21	2	1999	Midfielder	Primavara
23	2	2000	Midfielder	Primavara
24	2	1998	Midfielder	Primavara
25	2	1999	Midfielder	Primavara
25	2	1999	Midfielder	Primavara
26	2	1999	Midfielder	Primavara
27	2	2000	Midfielder	Primavara
1	3	2000	Midfielder	Primavara
3	3	1999	Midfielder	Primavara
4	3	2000	Midfielder	Primavara
5	3	1999	Midfielder	Primavara
7	3	2000	Midfielder	Primavara
7	3	2000	Midfielder	Primavara
8	3	1999	Midfielder	Primavara
9	3	1998	Midfielder	Primavara
10	3	1999	Midfielder	Primavara
10	3	1999	Midfielder	Primavara
11	3	2000	Midfielder	Primavara
12	3	1999	Midfielder	Primavara
12	3	1999	Midfielder	Primavara
13	3	2000	Midfielder	Primavara
16	3	1999	Midfielder	Primavara
19	3	1999	Midfielder	Primavara
20	3	2000	Midfielder	Primavara
22	3	1999	Midfielder	Primavara
23	3	1999	Midfielder	Primavara
25	3	1999	Midfielder	Primavara
26	3	2000	Midfielder	Primavara
26	3	1998	Midfielder	Primavara
30	3	1999	Midfielder	Primavara
31	3	2000	Midfielder	Primavara
2	4	1999	Midfielder	Primavara
3	4	2001	Midfielder	Primavara
9	4	1999	Midfielder	Primavara
9	4	1999	Midfielder	Primavara
12	4	2000	Midfielder	Primavara
12	4	1999	Midfielder	Primavara
12	4	1999	Midfielder	Primavara
12	4	2000	Midfielder	Primavara
16	4	2000	Midfielder	Primavara
16	4	2000	Midfielder	Primavara
20	4	2000	Midfielder	Primavara
25	4	2000	Midfielder	Primavara
28	4	2000	Midfielder	Primavara
29	4	2000	Midfielder	Primavara
30	4	1999	Midfielder	Primavara
3	5	1998	Midfielder	Primavara
5	5	2000	Midfielder	Primavara
11	5	1999	Midfielder	Primavara
15	5	1999	Midfielder	Primavara
17	5	1999	Midfielder	Primavara
19	5	1999	Midfielder	Primavara
22	5	1998	Midfielder	Primavara
23	5	1998	Midfielder	Primavara
26	5	1999	Midfielder	Primavara
27	5	2000	Midfielder	Primavara
30	5	1998	Midfielder	Primavara

DAY	MONTH	YEAR	ROLE	CATEGORY
30	5	1998	Midfielder	Primavara
2	6	2000	Midfielder	Primavara
3	6	1999	Midfielder	Primavara
12	6	2000	Midfielder	Primavara
15	6	1998	Midfielder	Primavara
19	6	1999	Midfielder	Primavara
21	6	1999	Midfielder	Primavara
24	6	1999	Midfielder	Primavara
26	6	1999	Midfielder	Primavara
27	6	1999	Midfielder	Primavara
30	6	2000	Midfielder	Primavara
2	7	1999	Midfielder	Primavara
4	7	1999	Midfielder	Primavara
5	7	1999	Midfielder	Primavara
13	7	1999	Midfielder	Primavara
15	7	1999	Midfielder	Primavara
18	7	1999	Midfielder	Primavara
19	7	1998	Midfielder	Primavara
20	7	1999	Midfielder	Primavara
25	7	1998	Midfielder	Primavara
27	7	1999	Midfielder	Primavara
28	7	1999	Midfielder	Primavara
29	7	1999	Midfielder	Primavara
31	7	1999	Midfielder	Primavara
2	8	1998	Midfielder	Primavara
6	8	2000	Midfielder	Primavara
9	8	2000	Midfielder	Primavara
10	8	1999	Midfielder	Primavara
12	8	1999	Midfielder	Primavara
19	8	1998	Midfielder	Primavara
22	8	2000	Midfielder	Primavara
26	8	1999	Midfielder	Primavara
28	8	1998	Midfielder	Primavara
29	8	2000	Midfielder	Primavara
30	8	1999	Midfielder	Primavara
31	8	1999	Midfielder	Primavara
4	9	1999	Midfielder	Primavara
5	9	1998	Midfielder	Primavara
12	9	1999	Midfielder	Primavara
18	9	1998	Midfielder	Primavara
27	9	2000	Midfielder	Primavara
29	9	1999	Midfielder	Primavara
1	10	1999	Midfielder	Primavara
2	10	2000	Midfielder	Primavara
3	10	2000	Midfielder	Primavara
10	10	1999	Midfielder	Primavara
15	10	1999	Midfielder	Primavara
21	10	1999	Midfielder	Primavara
25	11	1999	Midfielder	Primavara
30	11	1999	Midfielder	Primavara
2	12	2000	Midfielder	Primavara
4	12	1999	Midfielder	Primavara
7	12	2000	Midfielder	Primavara
9	12	1999	Midfielder	Primavara
22	12	1999	Midfielder	Primavara
23	12	1999	Midfielder	Primavara
31	12	1998	Midfielder	Primavara
1	1	1998	Defender	Serie A
2	1	1994	Defender	Serie A
2	1	1991	Defender	Serie A
5	1	1990	Defender	Serie A
7	1	1991	Defender	Serie A
7	1	1987	Defender	Serie A
9	1	1985	Defender	Serie A
9	1	1994	Defender	Serie A
12	1	1995	Defender	Serie A
13	1	1998	Defender	Serie A
13	1	1998	Defender	Serie A
16	1	1984	Defender	Serie A
17	1	1988	Defender	Serie A
17	1	1990	Defender	Serie A
20	1	1996	Defender	Serie A
20	1	1984	Defender	Serie A
20	1	1995	Defender	Serie A
22	1	1992	Defender	Serie A
26	1	1991	Defender	Serie A
28	1	1986	Defender	Serie A
30	1	1996	Defender	Serie A
1	2	1986	Defender	Serie A
1	2	1991	Defender	Serie A
1	2	1997	Defender	Serie A
2	2	1998	Defender	Serie A

DAY	MONTH	YEAR	ROLE	CATEGORY
2	2	1994	Defender	Serie A
3	2	1995	Defender	Serie A
3	2	1988	Defender	Serie A
5	2	1992	Defender	Serie A
5	2	1986	Defender	Serie A
5	2	1997	Defender	Serie A
6	2	1992	Defender	Serie A
8	2	1988	Defender	Serie A
8	2	1998	Defender	Serie A
8	2	2000	Defender	Serie A
10	2	1988	Defender	Serie A
10	2	1994	Defender	Serie A
11	2	1987	Defender	Serie A
11	2	1995	Defender	Serie A
11	2	1982	Defender	Serie A
11	2	1995	Defender	Serie A
11	2	1987	Defender	Serie A
15	2	1992	Defender	Serie A
15	2	1986	Defender	Serie A
16	2	1988	Defender	Serie A
18	2	1989	Defender	Serie A
19	2	1993	Defender	Serie A
20	2	1983	Defender	Serie A
21	2	1991	Defender	Serie A
29	2	1988	Defender	Serie A
1	3	1990	Defender	Serie A
1	3	1999	Defender	Serie A
2	3	1994	Defender	Serie A
2	3	1992	Defender	Serie A
5	3	1987	Defender	Serie A
5	3	1992	Defender	Serie A
7	3	1993	Defender	Serie A
17	3	1993	Defender	Serie A
17	3	1987	Defender	Serie A
18	3	1995	Defender	Serie A
21	3	1997	Defender	Serie A
22	3	1997	Defender	Serie A
27	3	1990	Defender	Serie A
30	3	1998	Defender	Serie A
3	4	1985	Defender	Serie A
7	4	1987	Defender	Serie A
12	4	1981	Defender	Serie A
12	4	1995	Defender	Serie A
12	4	1999	Defender	Serie A
13	4	1999	Defender	Serie A
15	4	1988	Defender	Serie A
16	4	1996	Defender	Serie A
17	4	1987	Defender	Serie A
17	4	1996	Defender	Serie A
17	4	1988	Defender	Serie A
17	4	1993	Defender	Serie A
19	4	1992	Defender	Serie A
21	4	1993	Defender	Serie A
22	4	1993	Defender	Serie A
22	4	1988	Defender	Serie A
25	4	1994	Defender	Serie A
28	4	1989	Defender	Serie A
1	5	1987	Defender	Serie A
1	5	1991	Defender	Serie A
4	5	1990	Defender	Serie A
5	5	1997	Defender	Serie A
5	5	1994	Defender	Serie A
5	5	1992	Defender	Serie A
6	5	1998	Defender	Serie A
8	5	1981	Defender	Serie A
9	5	1985	Defender	Serie A
10	5	1984	Defender	Serie A
11	5	1992	Defender	Serie A
12	5	1990	Defender	Serie A
13	5	1991	Defender	Serie A
17	5	1987	Defender	Serie A
19	5	1996	Defender	Serie A
20	5	1991	Defender	Serie A
21	5	1995	Defender	Serie A
22	5	1987	Defender	Serie A
23	5	1988	Defender	Serie A
25	5	1993	Defender	Serie A
26	5	1997	Defender	Serie A
26	5	1994	Defender	Serie A
27	5	1991	Defender	Serie A
31	5	1996	Defender	Serie A
5	6	1994	Defender	Serie A

DAY	MONTH	YEAR	ROLE	CATEGORY
6	6	1993	Defender	Serie A
9	6	1979	Defender	Serie A
10	6	1986	Defender	Serie A
10	6	1991	Defender	Serie A
10	6	1989	Defender	Serie A
10	6	1985	Defender	Serie A
11	6	1981	Defender	Serie A
14	6	1991	Defender	Serie A
17	6	1991	Defender	Serie A
20	6	1991	Defender	Serie A
21	6	1997	Defender	Serie A
26	6	1981	Defender	Serie A
27	6	1991	Defender	Serie A
29	6	1990	Defender	Serie A
30	6	1990	Defender	Serie A
1	7	1994	Defender	Serie A
2	7	1994	Defender	Serie A
3	7	1988	Defender	Serie A
5	7	1994	Defender	Serie A
9	7	1982	Defender	Serie A
12	7	1992	Defender	Serie A
17	7	1992	Defender	Serie A
17	7	1987	Defender	Serie A
22	7	1985	Defender	Serie A
25	7	1994	Defender	Serie A
29	7	1994	Defender	Serie A
29	7	1999	Defender	Serie A
30	7	1983	Defender	Serie A
31	7	1984	Defender	Serie A
2	8	1994	Defender	Serie A
3	8	1994	Defender	Serie A
3	8	1982	Defender	Serie A
3	8	1994	Defender	Serie A
11	8	1989	Defender	Serie A
13	8	1997	Defender	Serie A
14	8	1984	Defender	Serie A
17	8	1997	Defender	Serie A
18	8	1984	Defender	Serie A
25	8	1992	Defender	Serie A
26	8	1990	Defender	Serie A
27	8	1981	Defender	Serie A
30	8	1987	Defender	Serie A
1	9	1992	Defender	Serie A
4	9	1985	Defender	Serie A
7	9	1984	Defender	Serie A
8	9	1993	Defender	Serie A
9	9	1990	Defender	Serie A
9	9	1988	Defender	Serie A
10	9	1997	Defender	Serie A
12	9	1986	Defender	Serie A
12	9	1994	Defender	Serie A
20	9	1986	Defender	Serie A
20	9	1988	Defender	Serie A
25	9	1993	Defender	Serie A
25	9	1987	Defender	Serie A
25	9	1984	Defender	Serie A
30	9	1986	Defender	Serie A
2	10	1994	Defender	Serie A
6	10	1992	Defender	Serie A
8	10	1986	Defender	Serie A
10	10	1990	Defender	Serie A
12	10	1997	Defender	Serie A
14	10	1991	Defender	Serie A
14	10	1994	Defender	Serie A
20	10	1992	Defender	Serie A
21	10	1999	Defender	Serie A
22	10	1986	Defender	Serie A
25	10	1991	Defender	Serie A
31	10	1980	Defender	Serie A
31	10	1992	Defender	Serie A
2	11	1993	Defender	Serie A
5	11	1986	Defender	Serie A
10	11	1985	Defender	Serie A
12	11	1986	Defender	Serie A
14	11	1989	Defender	Serie A
19	11	1994	Defender	Serie A
23	11	1991	Defender	Serie A
25	11	1991	Defender	Serie A
29	11	1997	Defender	Serie A
2	12	1986	Defender	Serie A
5	12	1995	Defender	Serie A
5	12	1991	Defender	Serie A

DAY	MONTH	YEAR	ROLE	CATEGORY
5	12	1994	Defender	Serie A
6	12	1996	Defender	Serie A
10	12	1993	Defender	Serie A
14	12	1985	Defender	Serie A
16	12	1994	Defender	Serie A
19	12	1993	Defender	Serie A
1	1	1999	Forward	Serie A
3	1	1998	Forward	Serie A
4	1	1990	Forward	Serie A
4	1	1990	Forward	Serie A
5	1	1988	Forward	Serie A
6	1	1998	Forward	Serie A
6	1	1987	Forward	Serie A
9	1	1997	Forward	Serie A
12	1	1998	Forward	Serie A
14	1	1993	Forward	Serie A
16	1	1996	Forward	Serie A
19	1	1993	Forward	Serie A
19	1	1998	Forward	Serie A
19	1	1996	Forward	Serie A
24	1	1997	Forward	Serie A
24	1	1996	Forward	Serie A
29	1	1997	Forward	Serie A
31	1	1983	Forward	Serie A
2	2	1997	Forward	Serie A
3	2	1997	Forward	Serie A
5	2	1982	Forward	Serie A
7	2	1990	Forward	Serie A
11	2	1994	Forward	Serie A
11	2	1987	Forward	Serie A
15	2	1988	Forward	Serie A
16	2	1994	Forward	Serie A
18	2	1998	Forward	Serie A
19	2	1993	Forward	Serie A
20	2	1990	Forward	Serie A
25	2	1993	Forward	Serie A
27	2	1993	Forward	Serie A
28	2	1996	Forward	Serie A
28	2	2000	Forward	Serie A
28	2	1994	Forward	Serie A
6	3	1992	Forward	Serie A
11	3	1999	Forward	Serie A
14	3	1997	Forward	Serie A
15	3	1996	Forward	Serie A
16	3	1993	Forward	Serie A
17	3	1993	Forward	Serie A
17	3	1999	Forward	Serie A
17	3	1986	Forward	Serie A
17	3	2001	Forward	Serie A
20	3	1991	Forward	Serie A
23	3	1991	Forward	Serie A
25	3	1996	Forward	Serie A
27	3	1990	Forward	Serie A
27	3	1998	Forward	Serie A
29	3	1993	Forward	Serie A
1	4	1991	Forward	Serie A
3	4	1984	Forward	Serie A
8	4	1996	Forward	Serie A
12	4	1979	Forward	Serie A
17	4	1984	Forward	Serie A
23	4	1996	Forward	Serie A
24	4	1983	Forward	Serie A
26	4	1993	Forward	Serie A
28	4	1999	Forward	Serie A
3	5	1980	Forward	Serie A
6	5	1994	Forward	Serie A
6	5	1987	Forward	Serie A
6	5	1995	Forward	Serie A
7	5	1992	Forward	Serie A
10	5	1990	Forward	Serie A
12	5	1991	Forward	Serie A
12	5	1995	Forward	Serie A
19	5	1990	Forward	Serie A
19	5	1999	Forward	Serie A
20	5	1985	Forward	Serie A
20	5	1997	Forward	Serie A
21	5	1997	Forward	Serie A
21	5	1986	Forward	Serie A
27	5	1990	Forward	Serie A
27	5	1994	Forward	Serie A
28	5	1999	Forward	Serie A
28	5	1994	Forward	Serie A

DAY	MONTH	YEAR	ROLE	CATEGORY
4	6	1991	Forward	Serie A
14	6	1994	Forward	Serie A
17	6	1991	Forward	Serie A
17	6	1991	Forward	Serie A
18	6	1982	Forward	Serie A
20	6	1990	Forward	Serie A
20	6	1996	Forward	Serie A
26	6	1991	Forward	Serie A
30	6	1995	Forward	Serie A
4	7	1995	Forward	Serie A
5	7	1992	Forward	Serie A
5	7	1995	Forward	Serie A
7	7	1995	Forward	Serie A
8	7	1999	Forward	Serie A
8	7	1998	Forward	Serie A
9	7	1999	Forward	Serie A
12	7	1992	Forward	Serie A
17	7	1998	Forward	Serie A
22	7	1991	Forward	Serie A
23	7	1987	Forward	Serie A
27	7	1983	Forward	Serie A
30	7	1993	Forward	Serie A
1	8	1994	Forward	Serie A
2	8	1984	Forward	Serie A
3	8	1993	Forward	Serie A
6	8	1998	Forward	Serie A
7	8	1997	Forward	Serie A
10	8	1992	Forward	Serie A
11	8	1997	Forward	Serie A
19	8	1995	Forward	Serie A
19	8	1984	Forward	Serie A
5	9	1988	Forward	Serie A
8	9	1984	Forward	Serie A
16	9	1994	Forward	Serie A
28	9	1987	Forward	Serie A
29	9	1991	Forward	Serie A
29	9	1992	Forward	Serie A
1	10	1994	Forward	Serie A
1	10	2001	Forward	Serie A
9	10	1992	Forward	Serie A
11	10	1998	Forward	Serie A
15	10	1996	Forward	Serie A
27	10	1992	Forward	Serie A
28	10	1995	Forward	Serie A
29	10	1996	Forward	Serie A
6	11	1995	Forward	Serie A
6	11	1998	Forward	Serie A
10	11	1988	Forward	Serie A
12	11	1981	Forward	Serie A
12	11	1991	Forward	Serie A
13	11	1988	Forward	Serie A
15	11	1993	Forward	Serie A
15	11	1986	Forward	Serie A
17	11	1986	Forward	Serie A
19	11	1993	Forward	Serie A
26	11	1988	Forward	Serie A
3	12	1987	Forward	Serie A
10	12	1987	Forward	Serie A
19	12	1994	Forward	Serie A
20	12	1993	Forward	Serie A
1	1	1983	Goalkeeper	Serie A
3	1	1999	Goalkeeper	Serie A
4	1	1999	Goalkeeper	Serie A
4	1	1999	Goalkeeper	Serie A
5	1	1998	Goalkeeper	Serie A
7	1	1977	Goalkeeper	Serie A
9	1	1994	Goalkeeper	Serie A
12	1	1987	Goalkeeper	Serie A
13	1	2000	Goalkeeper	Serie A
17	1	1981	Goalkeeper	Serie A
18	1	1978	Goalkeeper	Serie A
26	1	1992	Goalkeeper	Serie A
27	1	1987	Goalkeeper	Serie A
28	1	1978	Goalkeeper	Serie A
7	2	1983	Goalkeeper	Serie A
20	2	1997	Goalkeeper	Serie A
23	2	1998	Goalkeeper	Serie A
25	2	1999	Goalkeeper	Serie A
25	2	1996	Goalkeeper	Serie A
25	2	1988	Goalkeeper	Serie A
29	2	1992	Goalkeeper	Serie A
2	3	1991	Goalkeeper	Serie A

DAY	MONTH	YEAR	ROLE	CATEGORY
6	3	1983	Goalkeeper	Serie A
10	3	1989	Goalkeeper	Serie A
15	3	1987	Goalkeeper	Serie A
16	3	1990	Goalkeeper	Serie A
18	3	1995	Goalkeeper	Serie A
19	3	1995	Goalkeeper	Serie A
22	3	1997	Goalkeeper	Serie A
25	3	1981	Goalkeeper	Serie A
27	3	1999	Goalkeeper	Serie A
28	3	1979	Goalkeeper	Serie A
5	4	1997	Goalkeeper	Serie A
12	4	1988	Goalkeeper	Serie A
18	4	1990	Goalkeeper	Serie A
27	4	1991	Goalkeeper	Serie A
5	5	1991	Goalkeeper	Serie A
8	5	1991	Goalkeeper	Serie A
10	5	1992	Goalkeeper	Serie A
25	5	1999	Goalkeeper	Serie A
31	5	1996	Goalkeeper	Serie A
3	6	1996	Goalkeeper	Serie A
6	6	1990	Goalkeeper	Serie A
6	6	1992	Goalkeeper	Serie A
7	6	1998	Goalkeeper	Serie A
10	6	1978	Goalkeeper	Serie A
21	6	1999	Goalkeeper	Serie A
28	6	1994	Goalkeeper	Serie A
1	7	2000	Goalkeeper	Serie A
7	7	1990	Goalkeeper	Serie A
8	7	1983	Goalkeeper	Serie A
14	7	1984	Goalkeeper	Serie A
14	7	1990	Goalkeeper	Serie A
7	8	1989	Goalkeeper	Serie A
8	8	1999	Goalkeeper	Serie A
19	8	1991	Goalkeeper	Serie A
19	8	1997	Goalkeeper	Serie A
20	8	1997	Goalkeeper	Serie A
30	8	1992	Goalkeeper	Serie A
31	8	1982	Goalkeeper	Serie A
5	9	1993	Goalkeeper	Serie A
27	9	1992	Goalkeeper	Serie A
2	10	1992	Goalkeeper	Serie A
10	10	1993	Goalkeeper	Serie A
25	10	1985	Goalkeeper	Serie A
9	11	1977	Goalkeeper	Serie A
10	11	1992	Goalkeeper	Serie A
11	11	1999	Goalkeeper	Serie A
12	11	1983	Goalkeeper	Serie A
1	12	1985	Goalkeeper	Serie A
1	1	1999	Midfielder	Serie A
5	1	1992	Midfielder	Serie A
8	1	1991	Midfielder	Serie A
8	1	1998	Midfielder	Serie A
9	1	1987	Midfielder	Serie A
10	1	1989	Midfielder	Serie A
11	1	1991	Midfielder	Serie A
11	1	1995	Midfielder	Serie A
11	1	1994	Midfielder	Serie A
12	1	1985	Midfielder	Serie A
12	1	1993	Midfielder	Serie A
13	1	1988	Midfielder	Serie A
15	1	1994	Midfielder	Serie A
18	1	1986	Midfielder	Serie A
18	1	1985	Midfielder	Serie A
19	1	1996	Midfielder	Serie A
19	1	1993	Midfielder	Serie A
19	1	1986	Midfielder	Serie A
20	1	1993	Midfielder	Serie A
23	1	1996	Midfielder	Serie A
24	1	1983	Midfielder	Serie A
25	1	1985	Midfielder	Serie A
28	1	1984	Midfielder	Serie A
29	1	1994	Midfielder	Serie A
29	1	1988	Midfielder	Serie A
30	1	1986	Midfielder	Serie A
30	1	1992	Midfielder	Serie A
2	2	1995	Midfielder	Serie A
2	2	1989	Midfielder	Serie A
3	2	1990	Midfielder	Serie A
6	2	1986	Midfielder	Serie A
7	2	1997	Midfielder	Serie A
7	2	1993	Midfielder	Serie A
8	2	1994	Midfielder	Serie A

DAY	MONTH	YEAR	ROLE	CATEGORY
11	2	1991	Midfielder	Serie A
11	2	1996	Midfielder	Serie A
12	2	1993	Midfielder	Serie A
13	2	1990	Midfielder	Serie A
15	2	1996	Midfielder	Serie A
15	2	1990	Midfielder	Serie A
16	2	1998	Midfielder	Serie A
17	2	1991	Midfielder	Serie A
18	2	1999	Midfielder	Serie A
25	2	1989	Midfielder	Serie A
26	2	1995	Midfielder	Serie A
27	2	1995	Midfielder	Serie A
28	2	1987	Midfielder	Serie A
29	2	1992	Midfielder	Serie A
1	3	1993	Midfielder	Serie A
3	3	1995	Midfielder	Serie A
7	3	1995	Midfielder	Serie A
9	3	1992	Midfielder	Serie A
9	3	1993	Midfielder	Serie A
10	3	1993	Midfielder	Serie A
10	3	1993	Midfielder	Serie A
10	3	1989	Midfielder	Serie A
11	3	1991	Midfielder	Serie A
12	3	1992	Midfielder	Serie A
13	3	1983	Midfielder	Serie A
18	3	1995	Midfielder	Serie A
18	3	1991	Midfielder	Serie A
18	3	1984	Midfielder	Serie A
20	3	1997	Midfielder	Serie A
24	3	1992	Midfielder	Serie A
25	3	1997	Midfielder	Serie A
25	3	1993	Midfielder	Serie A
25	3	1992	Midfielder	Serie A
27	3	1996	Midfielder	Serie A
28	3	1995	Midfielder	Serie A
29	3	1991	Midfielder	Serie A
30	3	1990	Midfielder	Serie A
2	4	1990	Midfielder	Serie A
2	4	1993	Midfielder	Serie A
4	4	1987	Midfielder	Serie A
5	4	1996	Midfielder	Serie A
7	4	1994	Midfielder	Serie A
9	4	1989	Midfielder	Serie A
9	4	1987	Midfielder	Serie A
12	4	1988	Midfielder	Serie A
12	4	2000	Midfielder	Serie A
14	4	1996	Midfielder	Serie A
15	4	1993	Midfielder	Serie A
15	4	1992	Midfielder	Serie A
19	4	1985	Midfielder	Serie A
21	4	1986	Midfielder	Serie A
24	4	1997	Midfielder	Serie A
24	4	1992	Midfielder	Serie A
24	4	1983	Midfielder	Serie A
28	4	2000	Midfielder	Serie A
4	5	1995	Midfielder	Serie A
4	5	1988	Midfielder	Serie A
5	5	1994	Midfielder	Serie A
5	5	1985	Midfielder	Serie A
6	5	1986	Midfielder	Serie A
7	5	1995	Midfielder	Serie A
10	5	1987	Midfielder	Serie A
10	5	1988	Midfielder	Serie A
11	5	1986	Midfielder	Serie A
11	5	1991	Midfielder	Serie A
14	5	1994	Midfielder	Serie A
16	5	1996	Midfielder	Serie A
19	5	1993	Midfielder	Serie A
20	5	1997	Midfielder	Serie A
20	5	1994	Midfielder	Serie A
22	5	1991	Midfielder	Serie A
23	5	1998	Midfielder	Serie A
23	5	1986	Midfielder	Serie A
24	5	1994	Midfielder	Serie A
24	5	1989	Midfielder	Serie A
26	5	1988	Midfielder	Serie A
27	5	1994	Midfielder	Serie A
30	5	1987	Midfielder	Serie A
5	6	1997	Midfielder	Serie A
5	6	1999	Midfielder	Serie A
7	6	1996	Midfielder	Serie A
14	6	1998	Midfielder	Serie A

DAY	MONTH	YEAR	ROLE	CATEGORY
16	6	1988	Midfielder	Serie A
16	6	1995	Midfielder	Serie A
17	6	1990	Midfielder	Serie A
17	6	1995	Midfielder	Serie A
19	6	1996	Midfielder	Serie A
19	6	1985	Midfielder	Serie A
20	6	1986	Midfielder	Serie A
24	6	1998	Midfielder	Serie A
29	6	1984	Midfielder	Serie A
29	6	1997	Midfielder	Serie A
1	7	1996	Midfielder	Serie A
2	7	1999	Midfielder	Serie A
14	7	1995	Midfielder	Serie A
14	7	1997	Midfielder	Serie A
15	7	1984	Midfielder	Serie A
16	7	1997	Midfielder	Serie A
17	7	1997	Midfielder	Serie A
19	7	1995	Midfielder	Serie A
19	7	1998	Midfielder	Serie A
24	7	1983	Midfielder	Serie A
26	7	1988	Midfielder	Serie A
27	7	1987	Midfielder	Serie A
1	8	1995	Midfielder	Serie A
4	8	1995	Midfielder	Serie A
5	8	1995	Midfielder	Serie A
6	8	1994	Midfielder	Serie A
7	8	1995	Midfielder	Serie A
7	8	1986	Midfielder	Serie A
9	8	1996	Midfielder	Serie A
11	8	1997	Midfielder	Serie A
11	8	1994	Midfielder	Serie A
14	8	1992	Midfielder	Serie A
17	8	1990	Midfielder	Serie A
22	8	1989	Midfielder	Serie A
23	8	1993	Midfielder	Serie A
24	8	1991	Midfielder	Serie A
29	8	1988	Midfielder	Serie A
5	9	1998	Midfielder	Serie A
5	9	1990	Midfielder	Serie A
6	9	1995	Midfielder	Serie A
6	9	1993	Midfielder	Serie A
14	9	1990	Midfielder	Serie A
15	9	1990	Midfielder	Serie A
20	9	1991	Midfielder	Serie A
22	9	1999	Midfielder	Serie A
24	9	1999	Midfielder	Serie A
26	9	1996	Midfielder	Serie A
28	9	1996	Midfielder	Serie A
28	9	1996	Midfielder	Serie A
29	9	1989	Midfielder	Serie A
30	9	1997	Midfielder	Serie A
1	10	1997	Midfielder	Serie A
5	10	1992	Midfielder	Serie A
7	10	1993	Midfielder	Serie A
12	10	1998	Midfielder	Serie A
12	10	1989	Midfielder	Serie A
17	10	1992	Midfielder	Serie A
18	10	1995	Midfielder	Serie A
20	10	1995	Midfielder	Serie A
22	10	1998	Midfielder	Serie A
25	10	1997	Midfielder	Serie A
29	10	1996	Midfielder	Serie A
11	11	1996	Midfielder	Serie A
11	11	1996	Midfielder	Serie A
12	11	1984	Midfielder	Serie A
12	11	1990	Midfielder	Serie A
15	11	1995	Midfielder	Serie A
16	11	1992	Midfielder	Serie A
28	11	1992	Midfielder	Serie A
29	11	1993	Midfielder	Serie A
2	12	1991	Midfielder	Serie A
2	12	1990	Midfielder	Serie A
3	12	1994	Midfielder	Serie A
7	12	1986	Midfielder	Serie A
7	12	1984	Midfielder	Serie A
9	12	1988	Midfielder	Serie A
11	12	1993	Midfielder	Serie A
12	12	1986	Midfielder	Serie A
18	12	1989	Midfielder	Serie A
19	12	1996	Midfielder	Serie A
20	12	1991	Midfielder	Serie A
21	12	1991	Midfielder	Serie A

DAY	MONTH	YEAR	ROLE	CATEGORY
31	12	1993	Midfielder	Serie A
